# Enhanced top‐down control of herbivore population growth on plants with impaired defences

**DOI:** 10.1111/1365-2435.14175

**Published:** 2022-09-21

**Authors:** Saioa Legarrea, Arne Janssen, Lin Dong, Joris J. Glas, Yvonne M. van Houten, Alessandra Scala, Merijn R. Kant

**Affiliations:** ^1^ Evolutionary and Population Biology Institute for Biodiversity and Ecosystem Dynamics (IBED) University of Amsterdam Amsterdam The Netherlands; ^2^ Departamento de Agricultura y Alimentación Universidad de la Rioja Logroño Spain; ^3^ Department of Entomology Federal University of Viçosa Viçosa Brazil; ^4^ Rijk Zwaan Breeding B.V. De Lier The Netherlands; ^5^ Koppert Biological Systems Berkel en Rodenrijs The Netherlands

**Keywords:** biological control, Eriophyidae, Phytoseiidae, plant defence, russet mite, Solanaceae, spider mite, trichomes

## Abstract

1. Herbivore densities can be regulated by bottom‐up and top‐down forces such as plant defences and natural enemies, respectively. These forces can interact with each other to increase plant protection against herbivores; however, how much complementarity exists between bottom‐up and top‐down forces still remains to be fully elucidated. Particularly, because plant defences can hinder natural enemies, how these interactions affect herbivore performance and dynamics remains elusive.
2. To address this topic, we performed laboratory and greenhouse bioassays with herbivorous mite pests and predatory mites on mutant tomato plants that lack defensive hairs on stems and leaves. Particularly, we investigated the behaviour and population dynamics of different phytophagous mite species in the absence and presence of predatory mites.
3. We show that predatory mites do not only perform better on tomatoes lacking defensive hairs but also that they can suppress herbivore densities better and faster on these hairless plants. Hence, top‐down control of herbivores by natural enemies more than compensated the reduced bottom‐up herbivore control by plant defences.
4. Our results lead to the counter‐intuitive insight that removing, instead of introducing, plant defence traits can result in superior protection against important pests through biological control.

Read the free Plain Language Summary for this article on the Journal blog.

## Graphical abstract

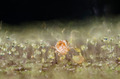

## 1 INTRODUCTION

Densities of herbivores can be suppressed by bottom‐up and top‐down forces, with bottom‐up forces mainly involving direct plant defences, consisting of a collection of physical and chemical barriers that decrease plant accessibility and that reduce its quality as food (reviewed in Kant et al., 2015). Top‐down forces mainly involve indirect plant defences (Halaj & Wise, 2001; Sabelis et al., 1999; Schmitz et al., 2000) consisting of attraction, arrestment and facilitation of natural enemies of herbivores to reduce damage caused by herbivory (Pearse et al., 2020; Price et al., 1980). Although there is a rich body of literature on plant defences increasing plant fitness (Erb, 2018), there are also frequent reports of plant defences failing, as is the case for plant trichomes (leaf hairs) that predominantly act as broad‐spectrum direct defence (reviewed in Glas et al., 2012), but occasionally fail to do so. For example, some herbivores evolved traits that enable them to feed on trichomes (Weinhold & Baldwin, 2011), to use trichomes to attach their silk to (Cardoso, 2008) or to use trichomes as a refuge (van Houten et al., 2013). Moreover, plant trichomes can also interfere with indirect defences (Ode, 2006; Price et al., 1980). There is ample literature on how chemical and physical plant traits such as thorns, wax covers of leaves or sticky leaf hairs can negatively affect natural enemies of herbivores (Eisner et al., 1998; Rabb & Bradley, 1968; Riddick & Simmons, 2014; Rutledge et al., 2003) thereby hampering top‐down control. This conflict between physicochemical plant defences and the efficiency of the natural enemies of herbivores is not only known from crop systems (Peterson et al., 2016; Riddick & Simmons, 2014) but also from natural tritrophic systems (Duffey & Bloem, 1986; Gassmann & Hare, 2005; Suzuki et al., 2011). Yet, some carnivorous insects evolved traits that enable them to cope with trichomes, for example, enabling them to move better on trichome‐rich surfaces (Voigt & Gorb, 2010), whereas other carnivores can even profit from them by feeding on insects trapped in sticky trichomes (Krimmel & Pearse, 2013). Overall, the wide‐spread occurrence of direct defences across the plant kingdom (Agrawal, 2007) suggests that natural selection in general does not act against it in favour of indirect defences.

Although there are examples of direct defences augmenting indirect defences (Krimmel & Pearse, 2013; Thaler, 1999), most studies indicate that especially constitutive plant defence traits, such as trichomes, can affect top‐down control negatively. Such defence traits can increase the mortality of the natural enemies such as coccinellids, crisopids, hoverflies (Belcher & Thurston, 1982; Elsey, 1974; Obrycky & Tauber, 1984; Verheggen et al., 2009), parasitoids (Kashyap et al., 1991; Keller, 1987; Romeis et al., 1999), and predatory mites (van Haren et al., 1987). These natural enemies are hampered by glandular trichomes, either through decreasing the density and quality of their herbivorous prey (Ataide et al., 2016; Duffey, 1980; Heidel‐Fischer & Vogel, 2015), or through decreasing their foraging efficiency by impeding predator movement (Carrillo et al., 2008; Fordyce & Agrawal, 2001; Krips et al., 1999; van Lenteren et al., 1995). Accordingly, predators were found to forage more efficiently in the absence of direct defences (Kaplan & Thaler, 2010; Kersch‐Becker & Thaler, 2015), but when direct defences are effective against herbivores, additional top‐down control appeared not to add much to this effect (Kersch‐Becker et al., 2017). Yet, what we do not know is to what extent top‐down control can compensate for failing bottom‐up control when plants are attacked by herbivores adapted to their direct defences.

The joint effect of plant defences and predators on the dynamics of herbivores has received relatively little attention; that is, most studies report effects over limited periods of time or as a seasonal average (Kaplan & Thaler, 2010; Kersch‐Becker & Thaler, 2015). This is not trivial because predator–prey dynamics often show fluctuations and populations that receive different treatments may do so with different frequencies (e.g. Coll & Ridgway, 1995) and thus peak at different moments. Depending on the time point at which the densities are evaluated, one could therefore find different effects of treatments on herbivore densities (see Janssen et al., 2006, for an example in a system with intraguild predation). Another factor affecting the densities of herbivores is the effect of the interaction between direct plant defences and predator population dynamics on the plants. Modelling approaches showed that predator mortality by external factors (modelled as caused by insecticides) results in higher pest densities, even when predator mortality due to these external factors was lower than that of the pest (Janssen & van Rijn, 2021). Similar arguments can be applied to mortality induced by direct plant defences, that is, pest densities in the presence of natural enemies may increase due to direct plant defences, even when the natural enemies suffer less from these defences than the pests. In this paper, we therefore investigate the effects of direct plant defences on herbivores and predator performance over time until plants without predators were overexploited by herbivores.

We hypothesized that the extent to which top‐down control can improve the protection of a plant depends on the extent to which the herbivores and their natural enemies are adapted to the plant's direct defences. We predicted that a reduction of direct defences (i.e. plant hairs) would increase herbivore performance, but would simultaneously promote the performance of natural enemies that suffer from the same defences, and the question then is, which of these two effects is predominant. To answer this question, we used a tritrophic model system that allows experimental manipulation of all these elements (see Figure S1 for an overview of the methodological approach). We used tomato plants because they possess a combination of physical and chemical defences in the form of glandular and nonglandular leaf hairs (trichomes) that contain sticky, toxic substances and therefore impede small leaf‐dwelling arthropod pests as well as predators (Duffey, 1986; Kennedy, 2003). In addition, well‐characterized tomato mutants are available that lack these trichomes (Stratmann & Bequette, 2016). As herbivores, we selected three mite species that differ in their degree of adaptation to tomato: one (*Tetranychus urticae* Koch, strain ‘Santpoort‐2’) is maladapted to tomato defences (Alba et al., 2015), another (*T. urticae* strain ‘Viçosa’) is an adapted tomato pest (Sarmento et al., 2011), and the last is a tomato specialist and important pest (*Aculops lycopersici* [Massee], Greenhalgh et al., 2020; Vervaet et al., 2021), which relies on tomato trichomes as a refuge (van Houten et al., 2013). Two phytoseiid mite species were chosen, which are potent control agents of the herbivores on several crops, but are severely hindered by tomato trichomes (Drukker et al., 1997; Krips et al., 1999; Paspati et al., 2021). Our results show that natural enemies do not only perform better on tomatoes lacking defensive hairs, but also suppress herbivore densities better and faster on these plants, with the largest effect being observed on plants infested with tomato‐adapted mites. We conclude that removing direct plant defences from crop plants, rather than reinforcing them, can be overcompensated by biological control, resulting in lower herbivore densities.

## 2 MATERIALS AND METHODS

### 2.1 Plant material and arthropod rearing

**FIGURE 1:**
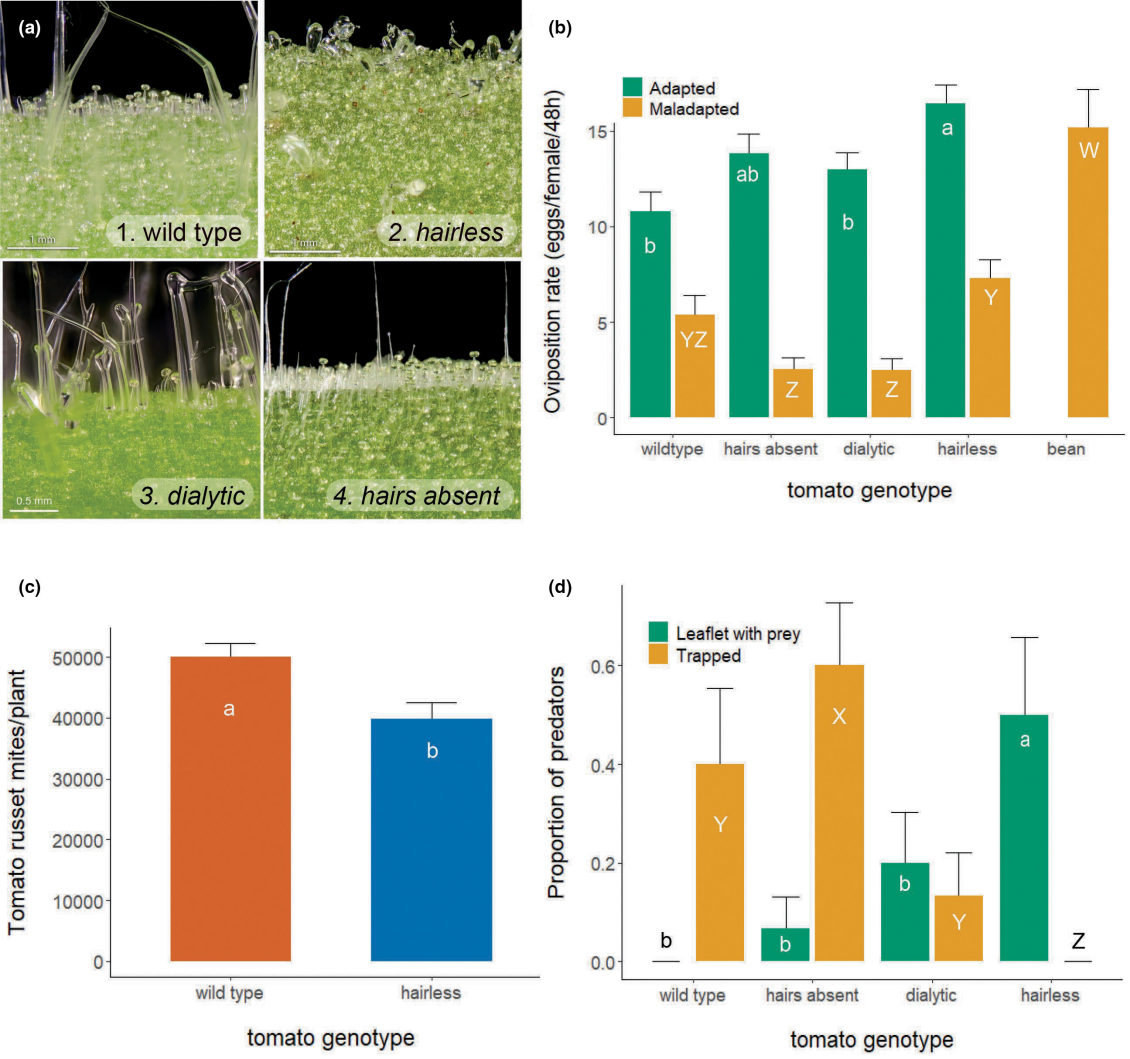
Trichome mutants and their effect on herbivore performance and predator movement. (a) Stem surface of wild type tomato and the trichome mutants *hairless, dialytic* and *hairs absent*. (b) Average oviposition rate (± SE) per 48 h of adapted (green bars) and maladapted (orange bars) spider mites on leaf discs of wild type tomato, trichome mutants and bean plants. Different letters signify significant differences for the maladapted (W‐Z) and adapted (a, b) spider mite strain (contrasts after LME, all *p* < 0.0001). (c) Average densities (± SE) of the tomato russet mite on wild type tomato plants and the *hairless* mutant 3 weeks after infestation. Letters in bars indicate significant differences (contrasts after GLM). (d) Proportion (± SE) of predators that was found trapped by the glandular trichomes (orange bars) or that reached a leaflet with prey (green bars) on wild type tomato and three trichome mutants. Letters Y, Z indicate significant differences between the proportions trapped, a, b indicates significant differences between the proportions that reached the leaflet with prey (contrasts after GLM, *p* < 0.05).

**FIGURE 2:**
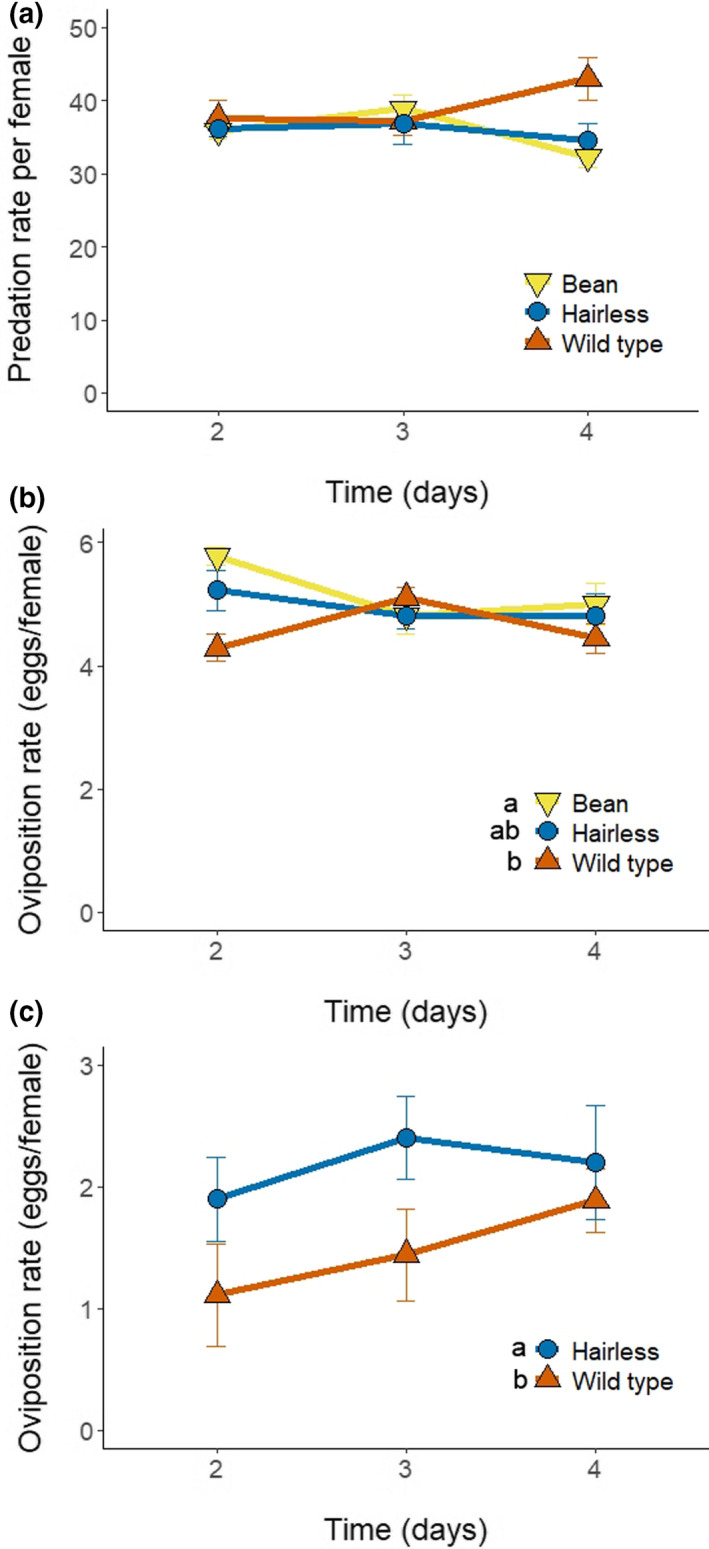
Effects of leaf trichomes on the performance of predatory mites on wild type (red) and *hairless* (blue) tomato leaf discs and on bean leaf discs (yellow). (a) Average predation rate (± SE) per 24 h of *P. persimilis* on bean, wild type or *hairless* tomato leaf discs infested with tomato‐adapted spider mites. (b) Average oviposition rate (± SE) per 24 h of *P. persimilis* when preying on the spider mites on the same leaf discs. (c) Average oviposition rate (± SE) per 24 h of *A. limonicus* when preying ad libitum on the tomato russet mite, placed on leaf disc of *hairless* and wild type tomato. Different letters signify significant differences (contrasts after LME, all *p* < 0.0001).

**FIGURE 3:**
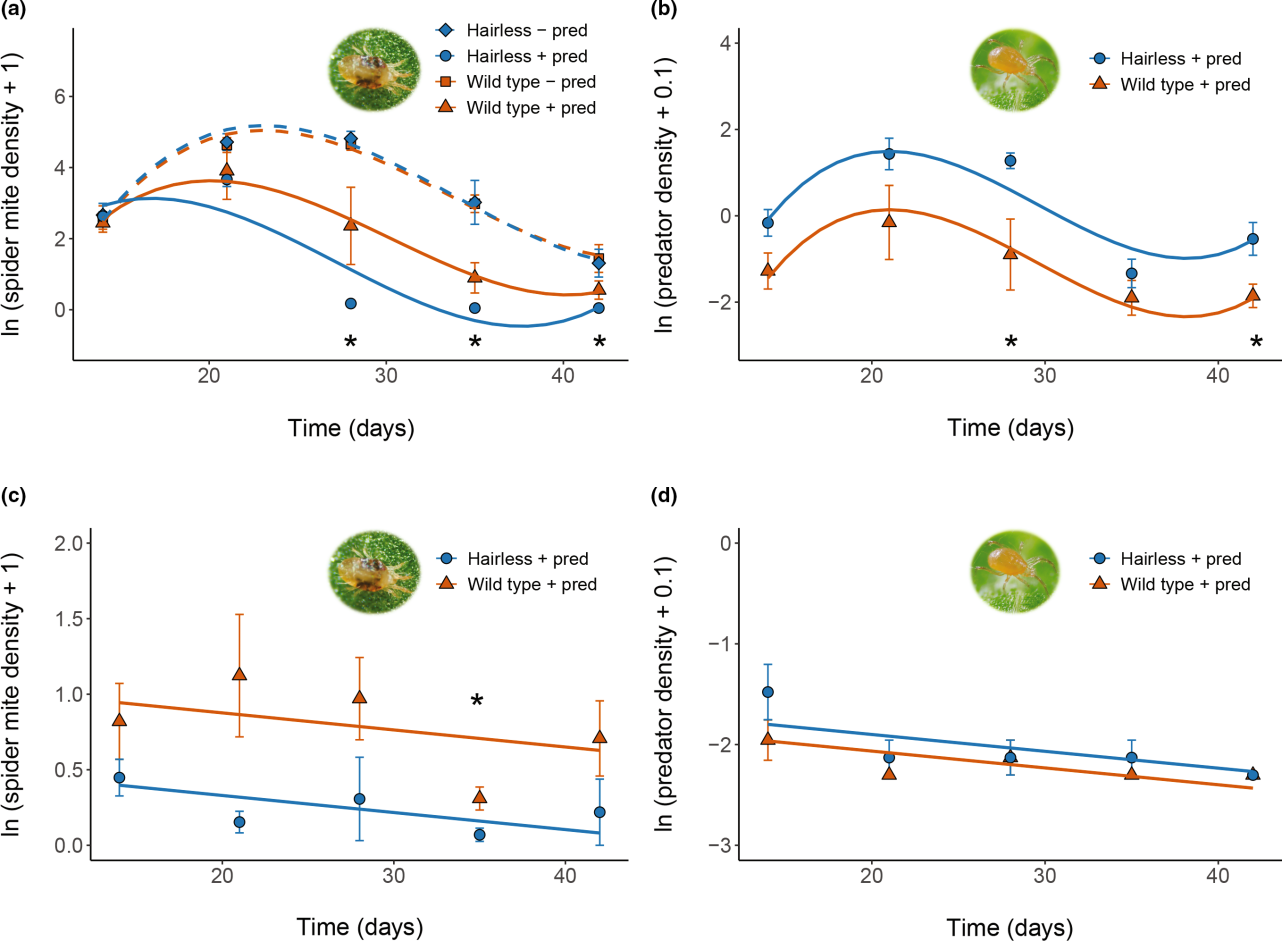
Spider mite and predatory mite population dynamics on wild type (red: Squares and triangles) and *hairless* (blue: Circles and diamonds) tomato plants. (a) Average log transformed densities (± SE) of adapted spider mites per leaflet without (dashed line) or with predators (solid line). (b) Average log transformed predator densities (± SE) on the same plants as in ‘a’. (c) Average log transformed densities (± SE) of maladapted spider mites on wild type and *hairless* plants with predatory mites. (d) Average log transformed predator densities (± SE) on the same plants as in ‘c’. All densities are per leaflet. Curves represent polynomial equations fit with a linear mixed‐effects model (LME). Asterisks indicate significant differences on specific dates based on glm analyses by date assuming a Gaussian distribution and identity link function.

**FIGURE 4:**
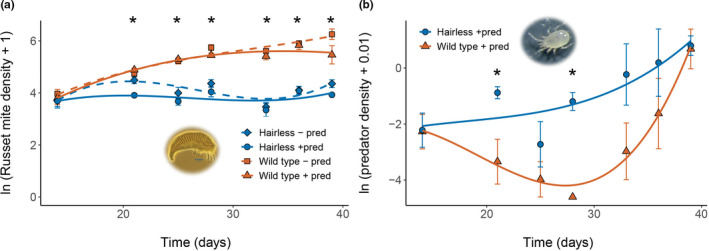
Russet mite and predatory mite population dynamics on wild type (red: Squares and triangles) and *hairless* (blue: Circles and diamonds) tomato. (a) Average log transformed densities (± SE) of the tomato russet mite with (solid line) or without (dashed line) predatory mites. (b) Average log‐transformed predator densities (± SE) on the same plants as in (a). All densities are expressed per leaflet. Curves are polynomial equations fit with a linear mixed‐effects model (LME). Asterisks indicate significant differences on specific dates based on glm analyses by date assuming a Gaussian distribution and identity link function.

We used *Solanum lycopersicum* L. cv. Ailsa Craig (TGRC accession #LA2838A) as wild type and several hair mutants in this wild type genetic background: *hairless* (accession #LA3556), *hairs absent* (TGRC accession #LA3172), and *dialytic* (TGRC accession #LA3724). Tomato seeds were obtained from the UC Davis/C.M. Rick Tomato Genetics Resource Center, maintained by the Department of Plant Sciences, University of California, Davis, CA 95616 (https://tgrc.ucdavis.edu/), where these mutants were developed. Plants were propagated at the University of Amsterdam greenhouse facilities. The wild type variety possesses glandular type I, VI and VII trichomes and nonglandular type III and V trichomes (Figure 1a‐1), common in cultivated tomato (Simmons & Gurr, 2005). The *hairless* (hl) accession (Kang et al., 2010) has morphological distortions of all trichome types: trichomes are twisted and swollen and often have a short stalk (Figure 1a‐2), whereas the density of glandular trichomes on leaves and stems is lower than that of the wild type (see Appendix S1). The *dialytic* (dl) accession (Chang et al., 2016) has forked trichomes with a different cellular arrangement and occurs at a lower density than wild type trichomes (Figure 1a‐3 and Appendix S1). Finally, the *hairs absent* (h) accession (Chang et al., 2018) does not have type I trichomes (Figure 1a‐4), and the density of glandular trichomes on leaves is lower than that of the wild type, while densities are similar on the stems (Appendix S1).

Laboratory assays were conducted using 4–6‐week‐old tomato plants, grown under controlled greenhouse conditions (20°C, 16:8 h [L:D] photoperiod) in 12 cm diameter pots (0.66 L) in a substrate consisting of peat soil (50% coco peat, 15% white peat, 35% frozen black peat; #3, Jongkind Grond BV, The Netherlands) without extra fertilizer. Different genotypes of tomato plants of the same age were placed together on the same greenhouse bench, occupying a surface of approximately one to two square meters to ensure that they were growing under the same environmental conditions. Bean (*Phaseolus vulgaris* L. cv. Speedy) plants were grown until 2–4‐week‐old under the same greenhouse conditions.

The maladapted strain of two‐spotted spider mites (*T. urticae* ‘Santpoort‐2’) was reared on detached bean leaves, and the adapted strain (*T. urticae* ‘Viçosa’) on detached leaves of wild type tomato for more than 25 generations. Tomato russet mites (*A. lycopersici*) were collected in 2008 from naturally infested plants in a greenhouse in the Netherlands and were maintained on intact tomato plants (cv. Castlemart) since then (Glas et al., 2014). The predatory mite *Phytoseiulus persimilis* Athias‐Henriot was provided by Alexandra Revynthi and had been maintained in the laboratory since 2013, reared on spider mites on bean leaves (Revynthi et al., 2018). A strain of *Amblydromalus limonicus* (Garman and McGregor) was provided by Koppert Biological Systems and maintained on plastic arenas with an ample supply of *Typha* pollen and access to water (Overmeer, 1985). The mite species were reared following standard techniques in climate rooms with controlled conditions (25°C, 16:8 h L:D, 60% RH).

### 2.2 Spider mite oviposition

Young spider mite females (15 ± 1 days old since egg) were individually placed on a leaf disc (2.4 cm diameter) with the abaxial side up on a layer of 1.5% Daishin Agar (Duchefa Biochemie bv, The Netherlands) and oviposition was recorded after 2 days. Oviposition was measured for both strains on wild type and trichome mutant leaf discs, and the strain from bean was additionally tested on bean leaf discs. The experiment was conducted in two blocks in time with 25 females per treatment in each block. Females that did not survive the 2‐day oviposition test were excluded from the analysis. The total oviposition during 2 days was compared among plants with a linear mixed effects model (LME, lme function of the package nlme; Pinheiro et al., 2020) with plant type/species and spider mite strain as fixed factors and block as a random factor. Contrasts were assessed with the Tukey method (*α* = 0.05) with the packages lsmeans (Lenth, 2016) and multcomp (Hothorn et al., 2008). Survival of spider mites was analysed with a generalized linear model with a quasi‐binomial error distribution and the same fixed factors as above. All models were checked for normality of the error distribution and all statistical analyses were done in R (version 3.3.3.) (R Core Team, 2017).

### 2.3 Russet mite performance

Three‐week‐old wild type and *hairless* plants were infested with 20 russet mite individuals using the method of van Houten et al. (2013). Briefly, individual mites were first transferred to a wild type leaf disc (1 cm diameter) using a stereomicroscope to make sure these minute fragile mites were not killed during the transfer. The infested leaf disc was subsequently placed on top of a leaflet of the oldest leaf of a plant using soft tweezers. As the leaf disc dried out, the mites moved to the plant. This leaf‐disc transfer method has been validated by means of a dose–response assay in Glas et al. (2014, figure S9). Twenty‐one days after the infestation, all plant material was collected and split into in four portions (leaves, including petioles, from the bottom, middle and top of the plant and the main stem), and samples were processed as described in Glas et al. (2014). In short, the plant material was cut into small pieces (i.e. leaflets or stem sections) and subsequently put in 50 ml tubes, to which 25 ml of 100% ethanol was added, and were shaken for 1 min to dislodge the mites from the plant surface. This process was repeated as needed to wash all plant material from each plant part in one 50 ml tube. Then, all samples were sieved (355 μm, Endecotts Ltd., UK) and mites in 1 ml of each tube were counted under a stereoscope. The total number of russet mites per plant was calculated. These densities of russet mites were analysed with a generalized linear model (GLM) with a Gaussian error distribution and plant genotype as fixed factor.

### 2.4 Predatory mite movement

The ability of predatory mites to move along the stems of the wild type and the trichome mutants was tested using the method of van Haren et al. (1987), slightly modified. All leaves except for two leaflets were removed from 5‐week‐old tomato plants and the leaflets were infested with 5 female adapted *T. urticae* for 2 days, serving as target sites for the predators. Two rings of glue (Tanglefoot®) were applied around the stem above and below these infested leaflets to confine predators. Per plant, a single gravid female *P. persimilis* was released on the stem between the two leaflets on the scar of one of the removed leaves. Plants were placed at random positions in cages (five plants per cage) and kept in climate rooms with controlled conditions (25°C, 16:8 h L:D, 60% RH). Twenty‐four hours later, the positions of the predatory mites, which could move freely upwards or downwards from the release point, were recorded under a stereo microscope. Predatory mites were scored as (1) being trapped when stuck on the glandular trichomes of the stem (‘Trapped’, Figure 1d), as (2) having reached the leaflet with prey (‘Leaflet with prey’, Figure 1d) or (3) as ‘other’ (not in the figure) when found back at the release point; in the glue barriers or on the stem. The group ‘other’ was excluded from the analysis. This resulted in 9, 8, 13 and 8 replicates for wild type plants, *hairless*, *hairs absent* and *dialytic*, respectively. The incidence of predators that were trapped or found on a leaflet with prey was evaluated among plant genotypes using a generalized linear model (GLM) in R (version 3.3.3., R Core Team, 2017) with a binomial error distribution. Planned contrasts among plant genotypes were assessed by aggregating nonsignificant factor levels in a stepwise deletion procedure (Crawley, 2013).

### 2.5 Predatory mite performance

To assess the effect of host plant on the performance of predatory mites, predation of eggs of the adapted strain of spider mites and oviposition by the predator *P. persimilis* were assessed on leaf discs (2.4 cm) on agar (as above) of *hairless* and wild type tomato and of bean plants. On each leaf disc, 5–15 spider mite females were allowed to lay eggs during 2 days (34–153 eggs). The adult females were subsequently removed, leaving their webbing intact to mimic a natural infestation of spider mites. Gravid female predators (judged by the size of their idiosoma) of 6 ± 1 days old since egg were released on these discs, and were transferred to a new arena with fresh spider mite eggs every day for four consecutive days. Predation and oviposition were recorded daily. The experiment was conducted in two blocks in time and had a total of 13–16 females per treatment. Data of the first day were excluded to avoid effects of the previous diet (Sabelis, 1990). Only replicates that had at least 10 prey eggs left after 24 h were included, ensuring that the predators had sufficient food. The average daily predation and oviposition of predatory mites were analysed with a linear mixed effects model (LME) as above, with plant type and time as fixed factors and predator individual as random factor to correct for repeated measures. Models were simplified by removing nonsignificant interactions and factors, and contrasts among plant genotypes were assessed as above.

The experimental arena for the russet mite predator *A. limonicus* was similar to that used for *P. persimilis*. A surplus (>100 individuals) of tomato russet mites from the rearing was added to the leaf disk with a one‐hair brush and they were allowed to settle for 1 day. Subsequently, an adult female *A. limonicus* of 9 ± 1 days old since egg was allowed to feed and lay eggs. Each predator was transferred daily to a new arena. Oviposition was recorded as the number of eggs laid per female every day of the experiment, excluding the first day. The experiment was conducted in two blocks in time for a total of 10 replicates per treatment. The average daily oviposition rate of predatory mites was analysed as above.

### 2.6 Dynamics of spider mites and predators

Cages (47.5 l × 47.5 w × 93 cm h) with two plants (either both *hairless* or both wild type) were distributed in a randomized block design on four parallel benches in a greenhouse at Rijk Zwaan (De Lier, The Netherlands). Plants regularly received water in the tray that held the pots of both plants. When the plants were 4 weeks old (3–4 true leaves), they were infested with 24 female spider mites (adapted or maladapted, 15 ± 1 days old since egg), 8 mites on a leaflet of leaves 1, 2 and 3, counting from the base of the plant. Young gravid females of *P. persimilis* were provided by Koppert Biological Systems (Berkel en Rodenrijs, The Netherlands) and were released 1 week later on a cotyledon of the plant (5 mites per plant). There were four treatments for the adapted strain: wild type or *hairless* plants either with or without predators, and two treatments for the maladapted strain: wild type or *hairless* plants both with predators, all treatments with four replicates (cages).

The dynamics of predators and prey were monitored by weekly sampling 3 leaflets from the top, middle and base of the plant (9 leaflets per cage). All mite stages were counted under a binocular microscope in the laboratory. For each species under evaluation (predator or prey), numbers of all stages were pooled to estimate population densities per leaflet. The experiment was terminated when plants without predators were overexploited by the spider mites. The moment of overexploitation was defined as when the 98%–100% of the leaves of wild type plants with spider mites but without predators were infested and all leaflets of a leaf showed clear signs of spider mite damage (leaf damage index 4 following Hussey & Scopes, 1985). The experiment was conducted during the winter (January–February), and the temperature was set to 25–18°C (day‐night), and the photoperiod was extended to 16 h with supplemental light.

Because the densities of spider mites and predators showed a highly nonlinear pattern through time, the average numbers of spider mites and predators per leaflet were log‐transformed. Furthermore, a polynomial was fit by adding quadratic and cubic terms of time (Crawley, 2013). Data were analysed using a linear mixed effects model (LME) with time, time squared and time cubed, all in interaction with treatment, as fixed factors. The replicate (cage) was used as a random factor to correct for repeated measures. An autocorrelation function of order 1 was added, but proved to be not significant and was removed. Models were simplified and contrasts among treatments were assessed in a stepwise deletion procedure of nonsignificant interactions and factors (Crawley, 2013). Additional analyses were conducted to gain insight into the specific dates in which treatments showed significant differences using generalized linear models (Gaussian distribution and an identity link function) followed by pairwise comparisons (adjusted with Tukey, *α* = 0.05) in R (version 4.2.0; 22 April 2022 ucrt) with the glm function and the package ‘emmeans’ (Lenth et al., 2019).

### 2.7 Dynamics of russet mites and predators

Experiments were carried out in cages as explained above (Dynamics of spider mites and predators). The four treatments consisted of russet mite‐infested wild type or *hairless* plants in the presence or absence of predatory mites (*A. limonicus*). Four‐week‐old plants were infested by placing leaf discs with 20–30 russet mites on one leaflet of leaves 1, 2 and 3, counting from the base of the stem (hence, 60–90 mites/plant). Seven days later, 10 adult female predatory mites were released on the leaflet of leaf 1 that was infested with prey. Bi‐weekly, three leaflets per plant stratum (as above) were collected in 50 ml falcon tubes and processed as described above with slight modifications. Briefly, 20 ml pure ethanol was added per tube in the lab, and mites were washed off the leaves by gently shaking tubes for 20 s. Samples were filtered through a sieve (160 μm) to collect and count predatory mites. Subsequently, the numbers of russet mites were assessed in five subsamples of 2 ml of the alcohol of each sample using a particle counter (PAMAS SVSS, Partikelmess‐ und Analysesysteme GmbH, Germany, size range 50–150 μm). The average of five particle counts was used for further analysis. The experiment was terminated when plants in cages without predatory mites had no more leaflets due to overexploitation by the russet mites. Log‐transformed densities of russet mites and predatory mites were analysed with a polynomial model and glm by date as above (dynamics of spider mites and predators).

## 3 RESULTS

### 3.1 Spider mite oviposition and russet mite performance

Oviposition of the adapted spider mite strain on tomato was higher than that of the maladapted strain (Figure 1b), but the two strains responded differentially to the various mutants (LME, interaction mite strain with plant type: Chi^2^ = 14.3, df = 3, *p* = 0.0025). Oviposition of the adapted spider mites differed significantly among plant types (LME: Chi^2^ = 19.7, df = 3, *p* < 0.001), and was higher on *hairless* than on the wild type and *dialytic*, and intermediate on *hairs absent* (Figure 1b). Oviposition of the maladapted strain also differed significantly among plant types (LME, Chi^2^ = 79.2, df = 4, *p* < 0.0001), was the highest on bean, the plant on which it was reared; intermediate on *hairless* and wild type; and lowest on *hairs absent* and *dialytic* (Figure 1b). Although the survival of the adapted strain was significantly higher than that of the maladapted strain (GLM, *F*_1,14_ = 8.07, *p* = 0.013), the survival of each strain did not differ significantly among plants (GLM, adapted strain: *F*_4,5_ = 0.84, *p* = 0.55; maladapted strain: *F*_5,6_ = 6.39, *p* = 0.28). In contrast, the population growth of the tomato russet mite was reduced on *hairless* tomato, relative to the wild type (Figure 1c, GLM: *F*_1,18_ = 8.34, *p* = 0.0098).

### 3.2 Predatory mite movement

The proportions of predators trapped in the trichomes differed significantly among plant types (GLM, Chi^2^ = 15.7, df = 3, *p* = 0.0013), as did the proportions that reached the target leaflet (GLM, Chi^2^ = 10.9, df = 3, *p* = 0.012) (Figure 1d). On the wild type, a high proportion of *P. persimilis* was trapped in the glandular trichomes and none reached the prey‐infested target leaflet. Similar results were obtained on the *hairs absent* mutant, and only c. 5% reached the leaflet with prey. On *dialytic*, the number of predators trapped was significantly reduced, yet only few reached the leaflet with prey. In contrast, no predatory mites were trapped on *hairless*, and a significant number of them reached the leaflet with prey in 24 h. We therefore selected the *hairless* accession for the following experiments. Finally, a predator was found back trapped in the Tanglefoot® only three times, that is, one on a *hairless*, one on a hairs absent and one on a wild type plant.

### 3.3 Predatory mite performance

Prey consumption rates by *P. persimilis* were similar on different plants (Figure 2a, LME, Chi^2^ = 1.85, df = 2, *p* = 0.40), although they produced more eggs on bean plants than on wild type tomato plants (Figure 2b, LME, Chi^2^ = 6.17, df = 2, *p* = 0.046). The oviposition rate of the predatory mite *A. limonicus*, a natural enemy of russet mites, was higher on *hairless* than on wild type plants (Figure 2c, LME, Chi^2^ = 3.94, df = 1, *p* = 0.047) and increased significantly through time (LME, Chi^2^ = 8.0, df = 1, *p* = 0.0047).

### 3.4 Dynamics of spider mites and predators

On tomato plants in a greenhouse, the density of adapted spider mites initially increased in all treatments (Figure 3a), then declined either because of predation (after c. 21 days in treatments with predators) or because plants became overexploited by spider mites (after c. 28 days) in treatments without predators. The densities of spider mites differed significantly among treatments through time (Figure 3a, LME, treatment with time: Chi^2^ = 34.8, df = 3, *p* < 0.0001, interaction of treatment with time^2^: Chi^2^ = 31.5, df = 3, *p* < 0.0001). In the absence of predators, densities of adapted spider mites on wild type and *hairless* plants did not differ significantly (Figure 3a). However, when predators were present, they controlled spider mites significantly better on *hairless* plants than on wild type plants (Figure 3a), coinciding with densities of predatory mites being significantly higher on *hairless* plants (Figure 3b, LME, Chi^2^ = 8.76, df = 1, *p* = 0.0031). Maladapted spider mites were also controlled much better by predatory mites on *hairless* plants (Figure 3c,d, LME, Chi^2^ = 8.84, df = 1, *p* = 0.003). Further detailed results by date are shown in Appendix S2. Hence, although *hairless* tomatoes were equally susceptible to adapted spider mites as wild type plants, they were better protected in the presence of predatory mites. Predators had a similar effect on the densities of maladapted spider mites, albeit at much lower absolute levels and consistently declined over time. At the end of the experiment (at 42 days), the leaf damage indices of *hairless* and wild type plants infested with adapted spider mites were 4.3 and 4.0, respectively, whereas those of the same treatments that included predatory mites were 2.8 and 2.5. The indices of *hairless* and wild type plants infested with maladapted spider mites was 1.3 and 2.0 at this time point.

### 3.5 Dynamics of russet mites and predators

In contrast to the spider mites, the russet mite populations grew larger on wild type plants than on *hairless* plants and their densities differed significantly over time between treatments through time (Figure 4a, LME, interaction of treatment with time^2^: Chi^2^ = 8.32, df = 3, *p* = 0.0399, interaction of treatment with time^3^: Chi^2^ = 9.06, df = 3, *p* = 0.029). While predatory mites reduced the russet mite densities on both wild type and *hairless* plants, the lowest russet mite densities were reached on *hairless* (Figure 4a). The densities of predatory mites differed significantly over time between treatments (Figure 4b, LME, interaction of treatment with time: Chi^2^ = 6.93, df = 1, *p* = 0.009, interaction of treatment with time^2^: Chi^2^ = 6.74, df = 1, *p* = 0.009), and densities were on average higher on *hairless* than on wild type plants. Further detailed results by date are shown in Appendix S2. Taken together, not only were *hairless* tomatoes more resistant to russet mites than the wild type, but the capacity of predatory mites to control them improved significantly on these plants (Figure 4).

## 4 DISCUSSION

We show that if a plant's direct defence fails because it is attacked by an adapted herbivore, its protection can be increased by replacing direct defences with top‐down control by natural enemies. We demonstrated this by using trichomeless tomatoes, on which the control efficiency of tomato‐adapted herbivorous mites by predatory mites is improved. Thus, counter‐intuitively, plants with high levels of direct defences may be rendered more vulnerable to herbivores; especially in cases when only herbivores, but not their natural enemies, have adapted to such defences.

Various studies have looked at the effects of direct plant defences on herbivore–predator interactions and have observed adverse effects of plant defences, such as trichomes, on natural enemies of herbivores (reviewed in: Peterson et al., 2016, Riddick & Simmons, 2014). However, there are only few studies that documented the predator–prey dynamics during such interactions (Barbour et al., 1993; Coll & Ridgway, 1995; Gassmann & Hare, 2005; Katanyukul & Thruston, 1973). Because predator–prey dynamics usually show fluctuations during their interaction on a plant (Figures 3 and 4), it is essential to study the dynamics over longer periods. Evidence for this in this study is the significant interaction of treatment with time. Had our experiment with adapted spider mites on plants lasted for 14 or 21 days, we would not have found a significant effect of treatments on spider mite densities, whereas we would have found significant treatment effects after 28, 35 or 42 days. Hence, the timing of the sampling of the densities matters, and even more so when populations fluctuate more strongly out of phase than the case here. We suggest that a natural end of population experiments like those presented here is when one of the interacting species has disappeared from the system. Here, we followed predator–prey populations until almost all leaf material was overexploited by herbivores on plants without predators.

Plant trichomes are widely recognized as relevant direct plant defences in both natural systems (Gassmann & Hare, 2005) and agricultural settings (Bleeker et al., 2012). For example, caterpillars gain more weight on plants with impaired glandular trichomes such as *hairless* (Kang et al., 2010; Tian et al., 2012); however, no effect of trichomes was found on weight of larvae of the Colorado potato beetle (Tian et al., 2012), and mites often get stuck in the sticky and toxic exudates of the glandular trichomes when present at high densities (Glas et al., 2012). Hence, it does not come as a surprise that tomato mutants like *hairless* are easier to handle for small leaf dwelling arthropods. Tomato plants of the *hairless* mutant do not only have distorted trichomes (Appendix S1), but also deficiencies in the chemistry of the glandular trichomes (i.e. sesquiterpenes and polyphenolic compounds) and an increase in stem brittleness (Kang et al., 2010, 2016). However, the expression of herbivore‐induced marker genes for jasmonate defences was equal or higher than that in the wild type (Tian et al., 2012). So, while the *hairless* mutant lacks the function of trichomes as physical and chemical barriers, the other (inducible) defences appear to be intact. The maladapted spider mite line used in our experiments induces jasmonate defences in tomato, from which it suffers (Alba et al., 2015). In line with this, we observed that their oviposition rate on trichome mutants was similar to that on wild type plants and much lower than that on bean. Kersch‐Becker et al. (2017) previously showed that biological control of aphids sensitive to jasmonate defences only had a minor additive effect to plant resistance on regulating herbivore population growth and final densities. In our experiments, the densities of maladapted herbivores on *hairless* plants were reduced significantly more by predatory mites than on wild type plants. However, their densities were much lower than that of the tomato‐adapted spider mites, and declined continuously from the start of the experiment. Crops are challenged by adapted and maladapted herbivores, yet, maladapted spider mites may also adapt to their host. *Tetranychus urticae* is a true generalist, found to colonize more than 1100 plant species (Migeon et al., 2021). A host switch such the one experienced in this study triggers large changes in spider mites in the long‐term (30 spider mite generations), including the overexpression of major detoxification pathways (Wybouw et al., 2015), but our experiments lasted too short to allow transcriptional plasticity and adaptation to occur. Therefore, direct defences may by themselves suffice to protect a crop from maladapted herbivores.

Whereas direct defences protect a plant against maladapted herbivores, they clearly fail for adapted herbivores. The tomato‐adapted spider mite used here is resistant to the plant's jasmonate defences (Sarmento et al., 2011). According to our data, it can also handle the tomato trichomes sufficiently, since its densities on *hairless* and wild type plants were similar. The densities of the adapted spider mites in the absence of predators started to decline at 28 days. Probably this was the result of a reduction of plant quality caused by herbivory and the consequent limitation of resources for the spider mites. When spider mites colonize a plant for a sufficient period of time, this can result in total damage of the leaves, resulting in the overexploitation of the host (Liu et al., 2017). Therefore, direct defences may not only fail when the plant is infested with adapted herbivores, but even hamper their control when they interfere with natural enemies of the herbivore.

Strikingly, the population growth of *A. lycopersici* in the absence of predators was even higher on wild type than on *hairless* plants, reinforcing the notion that direct (trichome‐based) defences are no universal panacea against all herbivores. Albeit speculatively, abiotic factors at the leaf surface, such as temperature or reflected radiation, are associated with the absence/presence of trichomes (Gasparini et al., 2021; Karabourniotis et al., 2020), which may result in an unfavourable microenvironment for this extremely small mite species on *hairless* plants. In addition to the tomato russet mite, other specialist herbivores of sticky plants have also shown decreased densities on genotypes that lack resistant glandular trichomes (Krimmel & Pearse, 2014; van Dam & Hare, 1998). Taken together, our data suggest that deficiencies in a plant's direct defence, such as those of the *hairless* mutant, make it more vulnerable to maladapted herbivores but not to adapted herbivores, whereas pest species will predominantly be found among the latter group.

Predatory mites were trapped on trichomes on the stem of wild type plants, thus supporting previous findings (Paspati et al., 2021; Sato et al., 2011; van Haren et al., 1987). However, our evaluation of the three trichome mutants suggests that a mere reduction of the trichome density may not be the only factor that improves predatory mite dispersal, but that reduced stickiness of glandular trichomes may also contribute to this. The glandular trichomes of *hairless* tomatoes contain a lower amount of phenolics than those of wild type plants (Kang et al., 2010), and this may contribute to a reduced entrapment since trichome stickiness is largely due to the oxidative formation of polyphenolics. In addition, we observed an increase in oviposition by *A. limonicus* on the *hairless* mutant. This suggests that especially stem trichomes affect the performance of predatory mites, but that some species are hampered by the trichomes on the leaves as well.

The predatory mites reached higher densities on *hairless* than on wild type plants when these were infested with adapted spider mites or the specialist tomato russet mites. On plants infested with maladapted mites, however, predatory mites experienced lack of food that limited the growth of the predator population. Yet, the predatory mites controlled the maladapted spider mites better on *hairless* than wild type plants, due to increased mobility on *hairless*, resulting in higher encounter rates with prey. Likewise, previous studies under laboratory conditions showed that phytoseiid mites increased their predation rates on host plants that lack disruptive glandular trichomes (Koller et al., 2007; Sato et al., 2011; Savi et al., 2021).

Our population dynamics experiments also have practical implications: they confirm not only that predatory mites are hindered by wild type trichomes but also that the disadvantage of promoting maladaptive herbivores by removing trichomes can be more than compensated by the enhanced control capacity of the predator. Phytoseiid mites are commonly used for biological control of various pests (Gerson & Weintraub, 2012; Knapp et al., 2018; van Lenteren, 2012), but biological control is cumbersome on tomato because of their trichomes (Cédola et al., 2001; van Haren et al., 1987; van Houten et al., 2013). Several attempts have been made to select for tomato‐adapted predators (Drukker et al., 1997; Lirakis & Magalhães, 2019), but this has not been very successful so far. Nowadays, only few natural enemies are known to cope with tomato glandular trichomes, such as mirid bugs (Bueno et al., 2019), and a few species of predatory mites (da Silva et al., 2010; Ferrero et al., 2011; Gigon et al., 2016), although the latter are still hindered by trichomes (Sato et al., 2011). Recently, it was found that large populations of predatory mites (i.e. Tydeoids), which are small enough to navigate under the trichome glands, can successfully control the tomato russet mite (Pijnakker et al., 2022). Current efforts in resistance breeding include developing cultivars that carry resistance traits against pests by reintroducing resistance traits, such as trichomes, from wild into commercial crop varieties (Glas et al., 2012; Paudel et al., 2019; Tissier, 2012). Our findings suggest that removing, rather than introducing, such traits may result in superior pest control by natural enemies.

Our results also justify the question to which extent plants in nature are under selection to adjust their direct defences to the needs of their putative bodyguards. Many plants have evolved glandular trichomes, perhaps because the efficiency of natural enemies is too low to sufficiently prevent herbivory. However, for reasons unknown, the majority of plants do not have such trichomes (Huchelmann et al., 2017), suggesting that other types of direct defences and/or indirect defences are sufficient. Furthermore, predators can also adapt to the direct defences of the host plants of their prey (Sun et al., 2019; Wheeler & Krimmel, 2015), in which case the direct defences become redundant for adapted herbivores, but may still serve to protect against other herbivores.

Taken together, our study emphasizes the importance of considering the effects of plant defence traits on the control of herbivores by their natural enemies. It also suggests novel opportunities for crop protection programs (Pappas et al., 2017; van Lenteren et al., 1995), because it not only warns breeders to be careful when introducing new resistance traits in crops, but also pleads for exploring the opposite approach of removing (unnecessary) resistance traits from crops embedded in integrated pest‐management programs.

## AUTHOR CONTRIBUTIONS

Merijn R. Kant, Arne Janssen and Saioa Legarrea conceived the original idea for this research. Saioa Legarrea, Lin Dong, Joris J. Glas, Yvonne M. van Houten and Alessandra Scala designed and conducted the experiments. Merijn R. Kant, Arne Janssen and Saioa Legarrea performed data analysis and wrote the manuscript. All authors revised the manuscript.

## CONFLICT OF INTEREST

The authors declare no conflict of interest.

## Supporting information


Appendix S1

Appendix S2

Figure S1

## Data Availability

The data supporting this manuscript is freely available at https://doi.org/10.6084/m9.figshare.20496231.v1 in figshare.com.

## References

[fec14175-bib-0001] Agrawal, A. A. (2007). Macroevolution of plant defense strategies. Trends in Ecology & Evolution, 22(2), 103–109. 10.1016/J.TREE.2006.10.012 17097760

[fec14175-bib-0002] Alba, J. M. , Schimmel, B. C. J. , Glas, J. J. , Ataide, L. M. S. , Pappas, M. L. , Villarroel, C. A. , Schuurink, R. C. , Sabelis, M. W. , & Kant, M. R. (2015). Spider mites suppress tomato defenses downstream of jasmonate and salicylate independently of hormonal crosstalk. The New Phytologist, 205(2), 828–840. 10.1111/NPH.13075 25297722 PMC4301184

[fec14175-bib-0003] Ataide, L. M. S. , Pappas, M. L. , Schimmel, B. C. J. , Lopez‐Orenes, A. , Alba, J. M. , Duarte, M. V. A. , Pallini, A. , Schuurink, R. C. , & Kant, M. R. (2016). Induced plant‐defenses suppress herbivore reproduction but also constrain predation of their offspring. Plant Science, 252, 300–310. 10.1016/J.PLANTSCI.2016.08.004 27717467

[fec14175-bib-0004] Barbour, J. D. , Farrar, R. R. , & Kennedy, G. G. (1993). Interaction of *Manduca sexta* resistance in tomato with insect predators of *Helicoverpa zea* . Entomologia Experimentalis et Applicata, 68(2), 143–155. 10.1111/j.1570-7458.1993.tb01697.x

[fec14175-bib-0005] Belcher, D. W. , & Thurston, R. (1982). Inhibition of movement of larvae of the convergent lady beetle by leaf trichomes of tobacco. Environmental Entomology, 11(1), 91–94. 10.1093/ee/11.1.91

[fec14175-bib-0006] Bleeker, P. M. , Mirabella, R. , Diergaarde, P. J. , VanDoorn, A. , Tissier, A. , Kant, M. R. , Prins, M. , de Vos, M. , Haring, M. A. , & Schuurink, R. C. (2012). Improved herbivore resistance in cultivated tomato with the sesquiterpene biosynthetic pathway from a wild relative. Proceedings of the National Academy of Sciences of the United States of America, 109(49), 20124–20129. 10.1073/PNAS.1208756109 23169639 PMC3523864

[fec14175-bib-0007] Bueno, V. H. P. , Lins, J. C. , Silva, D. B. , & van Lenteren, J. C. (2019). Is predation of *Tuta absoluta* by three neotropical mirid predators affected by tomato lines with different densities in glandular trichomes? Arthropod–Plant Interactions, 13(1), 41–48. 10.1007/s11829-018-9658-1

[fec14175-bib-0008] Cardoso, M. Z. (2008). Herbivore handling of a plants trichome: The case of *Heliconius charithonia* (L.) (Lepidoptera: Nymphalidae) and *Passiflora lobata* (Killip) hutch. (Passifloraceae). Neotropical Entomology, 37(3), 247–252. 10.1590/S1519-566X2008000300002 18641894

[fec14175-bib-0009] Carrillo, D. , Peña, J. E. , & Capinera, J. L. (2008). Effect of host plants on successful parasitism by *Haeckeliania sperata* (Hymenoptera: Trichogrammatidae) on *Diaprepes abbreviatus* (Coleoptera: Curculionidae) eggs. Environmental Entomology, 37(6), 1565–1572. 10.1603/0046-225X-37.6.1565 19161701

[fec14175-bib-0010] Cédola, C. v. , Sánchez, N. E. , & Liljesthröm, G. G. (2001). Effect of tomato leaf hairiness on functional and numerical response of *Neoseiulus californicus* (Acari: Phytoseiidae). Experimental and Applied Acarology, 25(10), 819–831. 10.1023/A:1020499624661 12455873

[fec14175-bib-0011] Chang, J. , Yu, T. , Gao, S. , Xiong, C. , Xie, Q. , Li, H. , Ye, Z. , & Yang, C. (2016). Fine mapping of the dialytic gene that controls multicellular trichome formation and stamen development in tomato. TAG. Theoretical and Applied Genetics. Theoretische Und Angewandte Genetik, 129(8), 1531–1539. 10.1007/S00122-016-2722-2 27151537

[fec14175-bib-0012] Chang, J. , Yu, T. , Yang, Q. , Li, C. , Xiong, C. , Gao, S. , Xie, Q. , Zheng, F. , Li, H. , Tian, Z. , Yang, C. , & Ye, Z. (2018). Hair, encoding a single C_2_H_2_ zinc‐finger protein, regulates multicellular trichome formation in tomato. The Plant Journal, 96(1), 90–102. 10.1111/TPJ.14018 29981215

[fec14175-bib-0013] Coll, M. , & Ridgway, R. L. (1995). Functional and numerical responses of *Orius insidiosus* (Heteroptera: Anthocoridae) to its prey in different vegetable crops. Annals of the Entomological Society of America, 88(6), 732–738. 10.1093/aesa/88.6.732

[fec14175-bib-0014] Crawley, M. J. (2013). The R book (2nd ed.). John Willey & Sons Ltd.

[fec14175-bib-0015] da Silva, F. R. , de Moraes, G. J. , Gondim, M. G., Jr. , Knapp, M. , Rouam, S. L. , Paes, J. L. , & de Oliveira, G. M. (2010). Efficiency of *Phytoseiulus longipes* Evans as a control agent of *Tetranychus evansi* Baker & Pritchard (Acari: Phytoseiidae: Tetranychidae) on screenhouse tomatoes. Neotropical Entomology, 39, 991–995. 10.1590/S1519-566X2010000600022 21271069

[fec14175-bib-0016] Drukker, B. , Janssen, A. , Ravensberg, W. , & Sabelis, M. W. (1997). Improved control capacity of the mite predator *Phytoseiulus persimilis* (Acari: Phytoseiidae) on tomato. Experimental and Applied Acarology, 21(6–7), 507–518. 10.1023/B:APPA.0000018885.35044.C6

[fec14175-bib-0017] Duffey, S. , & Bloem, K. (1986). Plant defense‐herbivore‐parasite interactions and biological control. In M. Kogan (Ed.), Ecological theory and integrated pest management practice (pp. 135–183). John Wiley & Sons Ltd.

[fec14175-bib-0018] Duffey, S. S. (1980). Sequestration of plant natural products by insects. Annual Review of Entomology, 25(1), 447–477. 10.1146/ANNUREV.EN.25.010180.002311

[fec14175-bib-0019] Duffey, S. S. (1986). Plant glandular trichomes: Their partial role in defence against insects. In B. Juniper & R. Southwood (Eds.), Insects and the plant surface (pp. 151–172). Edward Arnold Publishers.

[fec14175-bib-0020] Eisner, T. , Eisner, M. , & Hoebeke, E. R. (1998). When defense backfires: Detrimental effect of a plant's protective trichomes on an insect beneficial to the plant. Proceedings of the National Academy of Sciences of the United States of America, 95(8), 4410–4414. 10.1073/PNAS.95.8.4410 9539750 PMC22502

[fec14175-bib-0021] Elsey, K. D. (1974). Influence of plant host on searching speed of two predators. Entomophaga, 19(1), 3–6. 10.1007/BF02371503

[fec14175-bib-0022] Erb, M. (2018). Plant defenses against herbivory: Closing the fitness gap. Trends in Plant Science, 23(3), 187–194. 10.1016/J.TPLANTS.2017.11.005 29223923

[fec14175-bib-0023] Ferrero, M. , Calvo, F. J. , Atuahiva, T. , Tixier, M. S. , & Kreiter, S. (2011). Biological control of *Tetranychus evansi* Baker & Pritchard and *Tetranychus urticae* Koch by *Phytoseiulus longipes* Evans in tomato greenhouses in Spain [Acari: Tetranychidae, Phytoseiidae]. Biological Control, 58(1), 30–35. 10.1016/j.biocontrol.2011.03.012

[fec14175-bib-0024] Fordyce, J. A. , & Agrawal, A. A. (2001). The role of plant trichomes and caterpillar group size on growth and defence of the pipevine swallowtail *Battus philenor* . Journal of Animal Ecology, 70(6), 997–1005. 10.1046/J.0021-8790.2001.00568.X

[fec14175-bib-0025] Gasparini, K. , da Silva, M. F. , Costa, L. C. , Martins, S. C. , Ribeiro, D. M. , Peres, L. E. , & Zsögön, A. (2021). The *Lanata* trichome mutation increases stomatal conductance and reduces leaf temperature in tomato. Journal of Plant Physiology, 260, 153413. 10.1016/j.jplph.2021.153413 33848796

[fec14175-bib-0026] Gassmann, A. J. , & Hare, J. D. (2005). Indirect cost of a defensive trait: Variation in trichome type affects the natural enemies of herbivorous insects on *Datura wrightii* . Oecologia, 144(1), 62–71. 10.1007/S00442-005-0038-Z 15800744

[fec14175-bib-0027] Gerson, U. , & Weintraub, P. G. (2012). Mites (Acari) as a factor in greenhouse management. Annual Review of Entomology, 57, 229–247. 10.1146/annurev-ento-120710-100639 21910634

[fec14175-bib-0028] Gigon, V. , Camps, C. , & Le Corff, J. (2016). Biological control of *Tetranychus urticae* by *Phytoseiulus macropilis* and *Macrolophus pygmaeus* in tomato greenhouses. Experimental and Applied Acarology, 68(1), 55–70. 10.1007/s10493-015-9976-2 26481345

[fec14175-bib-0029] Glas, J. J. , Alba, J. M. , Simoni, S. , Villarroel, C. A. , Stoops, M. , Schimmel, B. C. J. , Schuurink, R. C. , Sabelis, M. W. , & Kant, M. R. (2014). Defense suppression benefits herbivores that have a monopoly on their feeding site but can backfire within natural communities. BMC Biology, 12, 1–14. 10.1186/S12915-014-0098-9 25403155 PMC4258945

[fec14175-bib-0030] Glas, J. J. , Schimmel, B. C. J. , Alba, J. M. , Escobar‐Bravo, R. , Schuurink, R. C. , & Kant, M. R. (2012). Plant glandular trichomes as targets for breeding or engineering of resistance to herbivores. International Journal of Molecular Sciences, 13, 17077–17103. 10.3390/ijms131217077 23235331 PMC3546740

[fec14175-bib-0031] Greenhalgh, R. , Dermauw, W. , Glas, J. J. , Rombauts, S. , Wybouw, N. , Thomas, J. , Alba, J. M. , Pritham, E. J. , Legarrea, S. , Feyereisen, R. , & Van de Peer, Y. (2020). Genome streamlining in a minute herbivore that manipulates its host plant. eLife, 9, e56689. 10.7554/eLife.56689 33095158 PMC7738191

[fec14175-bib-0032] Halaj, J. , & Wise, D. H. (2001). Terrestrial trophic cascades: How much do they trickle? The American Naturalist, 157(3), 262–281. 10.1086/319190 18707289

[fec14175-bib-0033] Heidel‐Fischer, H. M. , & Vogel, H. (2015). Molecular mechanisms of insect adaptation to plant secondary compounds. Current Opinion in Insect Science, 8, 8–14. 10.1016/J.COIS.2015.02.004 32846688

[fec14175-bib-0034] Hothorn, T. , Bretz, F. , & Westfall, P. (2008). Simultaneous inference in general parametric models. Biometrical Journal, 50(3), 346–363. 10.1002/bimj.200810425 18481363

[fec14175-bib-0035] Huchelmann, A. , Boutry, M. , & Hachez, C. (2017). Plant glandular trichomes: Natural cell factories of high biotechnological interest. Plant Physiology, 175(1), 6–22. 10.1104/PP.17.00727 28724619 PMC5580781

[fec14175-bib-0036] Hussey, N. W. , & Scopes, N. E. A. (1985). Greenhouse vegetables (Britain). In W. Helle & M. W. Sabelis (Eds.), Spider mites–their biology, natural enemies and control (Vol. 1B, pp. 285–298). Elsevier.

[fec14175-bib-0037] Janssen, A. , Montserrat, M. , Marc De Roos, A. , Hillerislambers, R. , de Roos, A. M. , Pallini, A. , & Sabelis, M. W. (2006). Intraguild predation usually does not disrupt biological control. In J. Brodeur & G. Boivin (Eds.), Trophic and guild interactions in biological control (pp. 21–44). Springer Netherlands. 10.1007/1-4020-4767-3_2

[fec14175-bib-0038] Janssen, A. , & van Rijn, P. C. (2021). Pesticides do not significantly reduce arthropod pest densities in the presence of natural enemies. Ecology Letters, 24(9), 2010–2024. 10.1111/ele.13819 34160871 PMC8453990

[fec14175-bib-0039] Kang, J. H. , Campos, M. L. , Zemelis‐Durfee, S. , Al‐Haddad, J. M. , Jones, A. D. , Telewski, F. W. , Brandizzi, F. , & Howe, G. A. (2016). Molecular cloning of the tomato *hairless* gene implicates Actin dynamics in trichome‐mediated defense and mechanical properties of stem tissue. Journal of Experimental Botany, 67(18), 5313–5324. 10.1093/JXB/ERW292 27481446 PMC5049383

[fec14175-bib-0040] Kang, J. H. , Shi, F. , Jones, A. D. , Marks, M. D. , & Howe, G. A. (2010). Distortion of trichome morphology by the hairless mutation of tomato affects leaf surface chemistry. Journal of Experimental Botany, 61(4), 1053–1064. 10.1093/JXB/ERP370 20018901 PMC2826649

[fec14175-bib-0041] Kant, M. R. , Jonckheere, W. , Knegt, B. , Lemos, F. , Liu, J. , Schimmel, B. C. J. , Villarroel, C. A. , Ataide, L. M. S. , Dermauw, W. , Glas, J. J. , Egas, M. , Janssen, A. , van Leeuwen, T. , Schuurink, R. C. , Sabelis, M. W. , & Alba, J. M. (2015). Mechanisms and ecological consequences of plant defence induction and suppression in herbivore communities. Annals of Botany, 115(7), 1015–1051. 10.1093/AOB/MCV054 26019168 PMC4648464

[fec14175-bib-0042] Kaplan, I. , & Thaler, J. S. (2010). Plant resistance attenuates the consumptive and non‐consumptive impacts of predators on prey. Oikos, 119(7), 1105–1113. 10.1111/J.1600-0706.2009.18311.X

[fec14175-bib-0043] Karabourniotis, G. , Liakopoulos, G. , Nikolopoulos, D. , & Bresta, P. (2020). Protective and defensive roles of non‐glandular trichomes against multiple stresses: Structure–function coordination. Journal of Forestry Research, 31(1), 1–12. 10.1007/s11676-019-01034-4

[fec14175-bib-0044] Kashyap, R. K. , Kennedy, G. G. , & Farrar, R. R. (1991). Behavioral response of *Trichogramma pretiosum* Riley and *Telenomus sphingis* (Ashmead) to trichome/methyl ketone mediated resistance in tomato. Journal of Chemical Ecology, 17(3), 543–556. 10.1007/BF00982125 24258805

[fec14175-bib-0045] Katanyukul, W. , & Thruston, R. (1973). Seasonal parasitism and predation of eggs of the tobacco hornworm on various host plants in Kentucky. Environmental Entomology, 2(5), 939–945. 10.1093/ee/2.5.939

[fec14175-bib-0046] Keller, M. A. (1987). Influence of leaf surfaces on movements by the hymenopterous parasitoid *Trichogramma exiguum* . Entomologia Experimentalis et Applicata, 43(1), 55–59. 10.1111/j.1570-7458.1987.tb02202.x

[fec14175-bib-0047] Kennedy, G. G. (2003). Tomato, pests, parasitoids, and predators: Tritrophic interactions involving the genus *Lycopersicon* . Annual Review of Entomology, 48, 51–72. 10.1146/ANNUREV.ENTO.48.091801.112733 12194909

[fec14175-bib-0048] Kersch‐Becker, M. F. , Kessler, A. , & Thaler, J. S. (2017). Plant defences limit herbivore population growth by changing predator–prey interactions. Proceedings of the Royal Society B: Biological Sciences, 284, 20171120. 10.1098/RSPB.2017.1120 PMC5597831 28878062

[fec14175-bib-0049] Kersch‐Becker, M. F. , & Thaler, J. S. (2015). Plant resistance reduces the strength of consumptive and non‐consumptive effects of predators on aphids. Journal of Animal Ecology, 84(5), 1222–1232. 10.1111/1365-2656.12371 25788108

[fec14175-bib-0050] Knapp, M. , van Houten, Y. , van Baal, E. , & Groot, T. (2018). Use of predatory mites in commercial biocontrol: Current status and future prospects. Acarologia, 58, 72–82. 10.24349/ACAROLOGIA/20184275

[fec14175-bib-0051] Koller, M. , Knapp, M. , & Schausberger, P. (2007). Direct and indirect adverse effects of tomato on the predatory mite *Neoseiulus californicus* feeding on the spider mite *Tetranychus evansi* . Entomologia Experimentalis et Applicata, 125(3), 297–305. 10.1111/j.1570-7458.2007.00625.x

[fec14175-bib-0052] Krimmel, B. A. , & Pearse, I. S. (2013). Sticky plant traps insects to enhance indirect defence. Ecology Letters, 16(2), 219–224. 10.1111/ele.12032 23205839

[fec14175-bib-0053] Krimmel, B. A. , & Pearse, I. S. (2014). Generalist and sticky plant specialist predators suppress herbivores on a sticky plant. Arthropod–Plant Interactions, 8(5), 403–410. 10.1007/s11829-014-9318-z

[fec14175-bib-0054] Krips, O. E. , Kleijn, P. W. , Willems, P. E. L. , Gols, G. J. Z. , & Dicke, M. (1999). Leaf hairs influence searching efficiency and predation rate of the predatory mite *Phytoseiulus persimilis* (Acari: Phytoseiidae). Experimental & Applied Acarology, 23(2), 119–131. 10.1023/A:1006098410165

[fec14175-bib-0055] Lenth, R. (2016). Least‐squares means: The R package lsmeans. Journal of Statistical Software, 69, 1–33. 10.18637/jss.v069.i01

[fec14175-bib-0056] Lenth, R. , Singmann, H. , Love, J. , Buerkner, P. , & Herve, M. (2019). Estimated marginal means, aka least‐squares means v. 1.1 . https://CRAN.R‐project.org/package=emmeans

[fec14175-bib-0057] Lirakis, M. , & Magalhães, S. (2019). Does experimental evolution produce better biological control agents? A critical review of the evidence. Entomologia Experimentalis et Applicata, 167(7), 584–597. 10.1111/EEA.12815

[fec14175-bib-0058] Liu, J. , Legarrea, S. , & Kant, M. R. (2017). Tomato reproductive success is equally affected by herbivores that induce or that suppress defenses. Frontiers in Plant Science, 8, 2128. 10.3389/FPLS.2017.02128 29326739 PMC5733352

[fec14175-bib-0059] Migeon, A. , Nouguier, E. , & Dorkeld, F. (2021). Spider mites web: A comprehensive database for the Tetranychidae . http://www1.montpellier.inra.fr/CBGP/spmweb

[fec14175-bib-0060] Obrycky, J. J. , & Tauber, M. J. (1984). Natural enemy activity on glandular pubescent potato plants in the greenhouse: An unreliable predictor of effects in the field. Environmental Entomology, 13(3), 679–683. 10.1093/ee/13.3.679

[fec14175-bib-0061] Ode, P. J. (2006). Plant chemistry and natural enemy fitness: Effects on herbivore and natural enemy interactions. Annual Review of Entomology, 51, 163–185. 10.1146/ANNUREV.ENTO.51.110104.151110 16332208

[fec14175-bib-0062] Overmeer, W. P. J. (1985). Rearing and handling. In W. Helle & M. W. Sabelis (Eds.), Spider mites, their biology, natural enemies and control (pp. 162–170). Elsevier.

[fec14175-bib-0063] Pappas, M. L. , Broekgaarden, C. , Broufas, G. D. , Kant, M. R. , Messelink, G. J. , Steppuhn, A. , Wäckers, F. , & van Dam, N. M. (2017). Induced plant defences in biological control of arthropod pests: A double‐edged sword. Pest Management Science, 73(9), 1780–1788. 10.1002/PS.4587 28387028 PMC5575458

[fec14175-bib-0064] Paspati, A. , Rambla, J. L. , López Gresa, M. P. , Arbona, V. , Gómez‐Cadenas, A. , Granell, A. , González‐Cabrera, J. , & Urbaneja, A. (2021). Tomato trichomes are deadly hurdles limiting the establishment of *Amblyseius swirskii* Athias‐Henriot (Acari: Phytoseiidae). Biological Control, 157, 104572. 10.1016/J.BIOCONTROL.2021.104572

[fec14175-bib-0065] Paudel, S. , Lin, P. A. , Foolad, M. R. , Ali, J. G. , Rajotte, E. G. , & Felton, G. W. (2019). Induced plant defenses against herbivory in cultivated and wild tomato. Journal of Chemical Ecology, 45(8), 693–707. 10.1007/S10886-019-01090-4/FIGURES/6 31367970

[fec14175-bib-0066] Pearse, I. S. , LoPresti, E. , Schaeffer, R. N. , Wetzel, W. C. , Mooney, K. A. , Ali, J. G. , Ode, P. J. , Eubanks, M. D. , Bronstein, J. L. , & Weber, M. G. (2020). Generalising indirect defence and resistance of plants. Ecology Letters, 23(7), 1137–1152. 10.1111/ELE.13512 32394591

[fec14175-bib-0067] Peterson, J. A. , Ode, P. J. , Oliveira‐Hofman, C. , & Harwood, J. D. (2016). Integration of plant defense traits with biological control of arthropod pests: Challenges and opportunities. Frontiers in Plant Science, 7, 1794. 10.3389/FPLS.2016.01794/BIBTEX 27965695 PMC5129739

[fec14175-bib-0068] Pijnakker, J. , Moerkens, R. , Vangansbeke, D. , Duarte, M. , Bellinkx, S. , Benavente, A. , Merckx, J. , Stevens, I. , & Wäckers, F. (2022). Dual protection: A tydeoid mite effectively controls both a problem pest and a key pathogen in tomato. Pest Management Science, 78(1), 355–361. 10.1002/ps.6647 34532955

[fec14175-bib-0069] Pinheiro, J. , Bates, D. , DebRoy, S. , Sarkar, D. , & R Core Team . (2020). nlme: linear and nonlinear mixed effects models (R package version 3.1–131.1). https://CRAN.R‐project.org/package=nlme

[fec14175-bib-0070] Price, P. W. , Bouton, C. E. , Gross, P. , McPheron, B. A. , Thompson, J. N. , & Weis, A. E. (1980). Interactions among three trophic levels: Influence of plants on interactions between insect herbivores and natural enemies. Annual Review of Ecology and Systematics, 11, 41–65. https://www.jstor.org/stable/2096902?seq=1

[fec14175-bib-0071] R Core Team . (2017). R: A language and environment for statistical computing. R Foundation for Statistical Computing. https://www.R‐project.org

[fec14175-bib-0072] Rabb, R. L. , & Bradley, J. R. (1968). The influence of host plants on parasitism of eggs of the tobacco hornworm. Journal of Economic Entomology, 61(5), 1249–1252. 10.1093/JEE/61.5.1249

[fec14175-bib-0073] Revynthi, A. M. , Egas, M. , Janssen, A. , & Sabelis, M. W. (2018). Prey exploitation and dispersal strategies vary among natural populations of a predatory mite. Ecology and Evolution, 8(21), 10384–10394. 10.1002/ECE3.4446 30464812 PMC6238141

[fec14175-bib-0074] Riddick, E. W. , & Simmons, A. M. (2014). Do plant trichomes cause more harm than good to predatory insects? Pest Management Science, 70(11), 1655–1665. 10.1002/PS.3772 24585676

[fec14175-bib-0075] Romeis, J. , Shanower, T. G. , & Zebitz, C. P. W. (1999). Why *Trichogramma* (Hymenoptera: Trichogrammatidae) egg parasitoids of *Helicoverpa armigera* (Lepidoptera: Noctuidae) fail on chickpea. Bulletin of Entomological Research, 89(1), 89–95. 10.1017/S0007485399000115

[fec14175-bib-0076] Rutledge, C. E. , Robinson, A. P. , & Eigenbrode, S. D. (2003). Effects of a simple plant morphological mutation on the arthropod community and the impacts of predators on a principal insect herbivore. Oecologia, 135(1), 39–50. 10.1007/s00442-002-1114-2 12647102

[fec14175-bib-0077] Sabelis, M. W. (1990). How to analyse prey preference when prey density varies? A new method to discriminate between effects of gut fullness and prey type composition. Oecologia, 82(3), 289–298. 10.1007/BF00317473 28312701

[fec14175-bib-0078] Sabelis, M. W. , van Baalen, M. , Bakker, F. M. , Bruin, J. , Drukker, B. , Egas, C. J. M. , Janssen, A. , Lesna, I. K. A. , Pels, S. H. , van Rijn, P. C. J. , & Scutareanu, P. (1999). The evolution of direct and indirect plant defence against herbivorous arthropods. In H. Olff , V. K. Brown , & R. H. Drent (Eds.), Herbivores: Between plants and predators (pp. 109–166). Blackwell.

[fec14175-bib-0079] Sarmento, R. A. , Lemos, F. , Bleeker, P. M. , Schuurink, R. C. , Pallini, A. , Oliveira, M. G. A. , Lima, E. R. , Kant, M. , Sabelis, M. W. , & Janssen, A. (2011). A herbivore that manipulates plant defence. Ecology Letters, 14(3), 229–236. 10.1111/J.1461-0248.2010.01575.X 21299823 PMC3084520

[fec14175-bib-0080] Sato, M. M. , de Moraes, G. J. , Haddad, M. L. , & Wekesa, V. W. (2011). Effect of trichomes on the predation of *Tetranychus urticae* (Acari: Tetranychidae) by *Phytoseiulus macropilis* (Acari: Phytoseiidae) on tomato, and the interference of webbing. Experimental and Applied Acarology, 54(1), 21–32. 10.1007/s10493-011-9426-8 21279537

[fec14175-bib-0081] Savi, P. J. , de Moraes, G. J. , & de Andrade, D. J. (2021). Effect of tomato genotypes with varying levels of susceptibility to *Tetranychus evansi* on performance and predation capacity of *Phytoseiulus longipes* . BioControl, 66(5), 687–700. 10.1007/s10526-021-10096-5

[fec14175-bib-0082] Schmitz, O. J. , Hambäck, P. A. , & Beckerman, A. P. (2000). Trophic cascades in terrestrial systems: A review of the effects of carnivore removals on plants. The American Naturalist, 155(2), 141–153. 10.1086/303311/ASSET/IMAGES/LARGE/FG2.JPEG 10686157

[fec14175-bib-0083] Simmons, A. T. , & Gurr, G. M. (2005). Trichomes of *Lycopersicon* species and their hybrids: Effects on pests and natural enemies. Agricultural and Forest Entomology, 7(4), 265–276. 10.1111/J.1461-9555.2005.00271.X

[fec14175-bib-0084] Stratmann, J. W. , & Bequette, C. J. (2016). Hairless but no longer clueless: Understanding glandular trichome development. Journal of Experimental Botany, 67(18), 5285–5287. 10.1093/JXB/ERW339 27703083 PMC5049401

[fec14175-bib-0085] Sun, R. , Jiang, X. , Reichelt, M. , Gershenzon, J. , Pandit, S. S. , & Vassão, D. G. (2019). Tritrophic metabolism of plant chemical defenses and its effects on herbivore and predator performance. eLife, 8, e51029. 10.7554/ELIFE.51029 31841109 PMC6934381

[fec14175-bib-0086] Suzuki, H. , Yasuda, K. , Ohashi, K. , Takahashi, H. , Fukaya, M. , Yano, S. , & Osakabe, M. (2011). Kanzawa spider mites acquire enemy‐free space on a detrimental host plant, oleander. Entomologia Experimentalis et Applicata, 138(3), 212–222. 10.1111/J.1570-7458.2010.01092.X

[fec14175-bib-0087] Thaler, J. S. (1999). Jasmonate‐inducible plant defences cause increased parasitism of herbivores. Nature, 399, 686–688. 10.1038/21420

[fec14175-bib-0088] Tian, D. , Tooker, J. , Peiffer, M. , Chung, S. H. , & Felton, G. W. (2012). Role of trichomes in defense against herbivores: Comparison of herbivore response to woolly and hairless trichome mutants in tomato (*Solanum lycopersicum*). Planta, 236(4), 1053–1066. 10.1007/S00425-012-1651-9 22552638

[fec14175-bib-0089] Tissier, A. (2012). Glandular trichomes: What comes after expressed sequence tags? The Plant Journal, 70(1), 51–68. 10.1111/J.1365-313X.2012.04913.X 22449043

[fec14175-bib-0090] van Dam, N. M. , & Hare, J. D. (1998). Differences in distribution and performance of two sap‐sucking herbivores on glandular and non‐glandular *Datura wrightii* . Ecological Entomology, 23(1), 22–32. 10.1046/j.1365-2311.1998.00110.x

[fec14175-bib-0091] van Haren, R. J. F. , Steenhuis, M. M. , Sabelis, M. W. , & de Ponti, O. M. B. (1987). Tomato stem trichomes and dispersal success of *Phytoseiulus persimilis* relative to its prey *Tetranychus urticae* . Experimental & Applied Acarology, 3(2), 115–121. 10.1007/BF01270473

[fec14175-bib-0092] van Houten, Y. M. , Glas, J. J. , Hoogerbrugge, H. , Rothe, J. , Bolckmans, K. J. F. , Simoni, S. , van Arkel, J. , Alba, J. M. , Kant, M. R. , & Sabelis, M. W. (2013). Herbivory‐associated degradation of tomato trichomes and its impact on biological control of *Aculops lycopersici* . Experimental and Applied Acarology, 60(2), 127–138. 10.1007/s10493-012-9638-6 23238958 PMC3641295

[fec14175-bib-0093] van Lenteren, J. C. (2012). The state of commercial augmentative biological control: Plenty of natural enemies, but a frustrating lack of uptake. BioControl, 57(1), 1–20. 10.1007/S10526-011-9395-1

[fec14175-bib-0094] van Lenteren, J. C. , Hua, L. Z. , Kamerman, J. W. , & Rumei, X. (1995). The parasite‐host relationship between *Encarsia Formosa* (Hym., Aphelinidae) and *Trialeurodes vaporariorum* (Hom., Aleyrodidae) XXVI. Leaf hairs reduce the capacity of *Encarsia* to control greenhouse whitefly on cucumber. Journal of Applied Entomology, 119(1–5), 553–559. 10.1111/J.1439-0418.1995.TB01335.X

[fec14175-bib-0095] Verheggen, F. J. , Capella, Q. , Schwartzberg, E. G. , Voigt, D. , & Haubruge, E. (2009). Tomato‐aphid‐hoverfly: A tritrophic interaction incompatible for pest management. Arthropod–Plant Interactions, 3(3), 141–149. 10.1007/s11829-009-9065-8

[fec14175-bib-0096] Vervaet, L. , De Vis, R. , De Clercq, P. , & Van Leeuwen, T. (2021). Is the emerging mite pest *Aculops lycopersici* controllable? Global and genome‐based insights in its biology and management. Pest Management Science, 77(6), 2635–2644. 10.1002/ps.6265 33415791

[fec14175-bib-0097] Voigt, D. , & Gorb, S. (2010). Locomotion in a sticky terrain. Arthropod–Plant Interactions, 4(2), 69–79. 10.1007/s11829-010-9088-1

[fec14175-bib-0098] Weinhold, A. , & Baldwin, I. T. (2011). Trichome‐derived O‐acyl sugars are a first meal for caterpillars that tags them for predation. Proceedings of the National Academy of Sciences of the United States of America, 108(19), 7855–7859. 10.1073/pnas.1101306108 21518882 PMC3093468

[fec14175-bib-0099] Wheeler, A. G. , & Krimmel, B. A. (2015). Mirid (Hemiptera: Heteroptera) specialists of sticky plants: Adaptations, interactions, and ecological implications. Annual Review of Entomology, 60, 393–414. 10.1146/ANNUREV-ENTO-010814-020932 25564742

[fec14175-bib-0100] Wybouw, N. , Zhurov, V. , Martel, C. , Bruinsma, K. A. , Hendrickx, F. , Grbic, V. , & van Leeuwen, T. (2015). Adaptation of a polyphagous herbivore to a novel host plant extensively shapes the transcriptome of herbivore and host. Molecular Ecology, 24(18), 4647–4663. 10.1111/MEC.13330 26211543

