# Supplementary material for: Enhanced top‐down control of herbivore population growth on plants with impaired defences
Source: Funct Ecol. 2022 Sep 21;36(11):2859–72. doi: 10.1111/1365-2435.14175 (PMC9826462; doi:10.1111/1365-2435.14175)
Supplement: Supplementary file 1 — Appendix S1 [file FEC-36-2859-s001.pdf]

Supporting Information for

Enhanced top-down control of herbivore population growth on plants with impaired defences

By: Legarrea S., Janssen A., Dong L., Glas J.J., van Houten Y.M., Scala A., & Kant M.R.

Published in Functional Ecology

Appendix S1: Trichome densities of wild type and trichome-mutant tomatoes

To assess trichome densities on leaves, plants were grown for 4 weeks. Six leaf discs (0.3 or 0.4 cm diameter) were cut from the fourth leaf from the top, avoiding the central vein of the leaf. Trichomes were counted on the upper side of three discs and on the underside of the other three discs. For the stem, plants were grown for 5 weeks and the stem between leaf number 4 and 5 from the top was delimited using a leaf puncher and trichomes were counted on three areas of the same diameter as above. Trichomes were classified as non-glandular (trichomes III and V) or glandular (trichomes I and VI), according to Glas et al., (2012), and counted using a stereomicroscope. Numbers were subsequently converted to densities per cm^2. For foliar trichomes, densities on upper side or underside were first averaged per leaflet and then per plant. For stem trichomes, data were averaged per plant stem. Average trichome densities per plant on the upper side of the leaves, on the underside and on the stem were analyzed separately. The numbers of plants were 4 for wild type, *hairless* and *hairs absent* and 2 for *dialytic*. Densities were analyzed with a GLM with a Gaussian error distribution. Contrasts among varieties were calculated with the lsmeans package of R (Lenth, 2016) with Tukey correction for multiple comparisons.

Compared to the wild type (AS1 Figure 1A), all mutants had lower trichome densities. The *hairless* genotype (AS1 Figure 1B) has distorted glandular and non-glandular trichomes and a different chemical profile of the glandular trichomes (according to Kang et al., 2010)), the *hairs absent* genotype (AS1 Figure 1C) has no trichomes of type I, and dialytic (AS1 Figure 1D) has branched non-glandular trichomes. Trichome densities on the upper side of leaves varied significantly among tomato varieties (AS1 Figure 1E, non-glandular trichomes: $F_{3,10}$ = 5.54, P = 0.017; glandular: $F_{3,10}$ = 15.5, P = 0.0004). Densities of non-glandular trichomes on the underside did not differ significantly among plant types (AS1 Figure 1F, $F_{3,10}$ = 3.65, P = 0.052), but densities of glandular hairs did ($F_{3,10}$ = 11.2, P = 0.0016). The same patterns as on the underside of the leaves was observed for the stems (AS1 Figure 1G, non-glandular: $F_{3,8}$ = 2.48, P = 0.14; glandular: $F_{3,8}$ = 9.85, P = 0.0046).

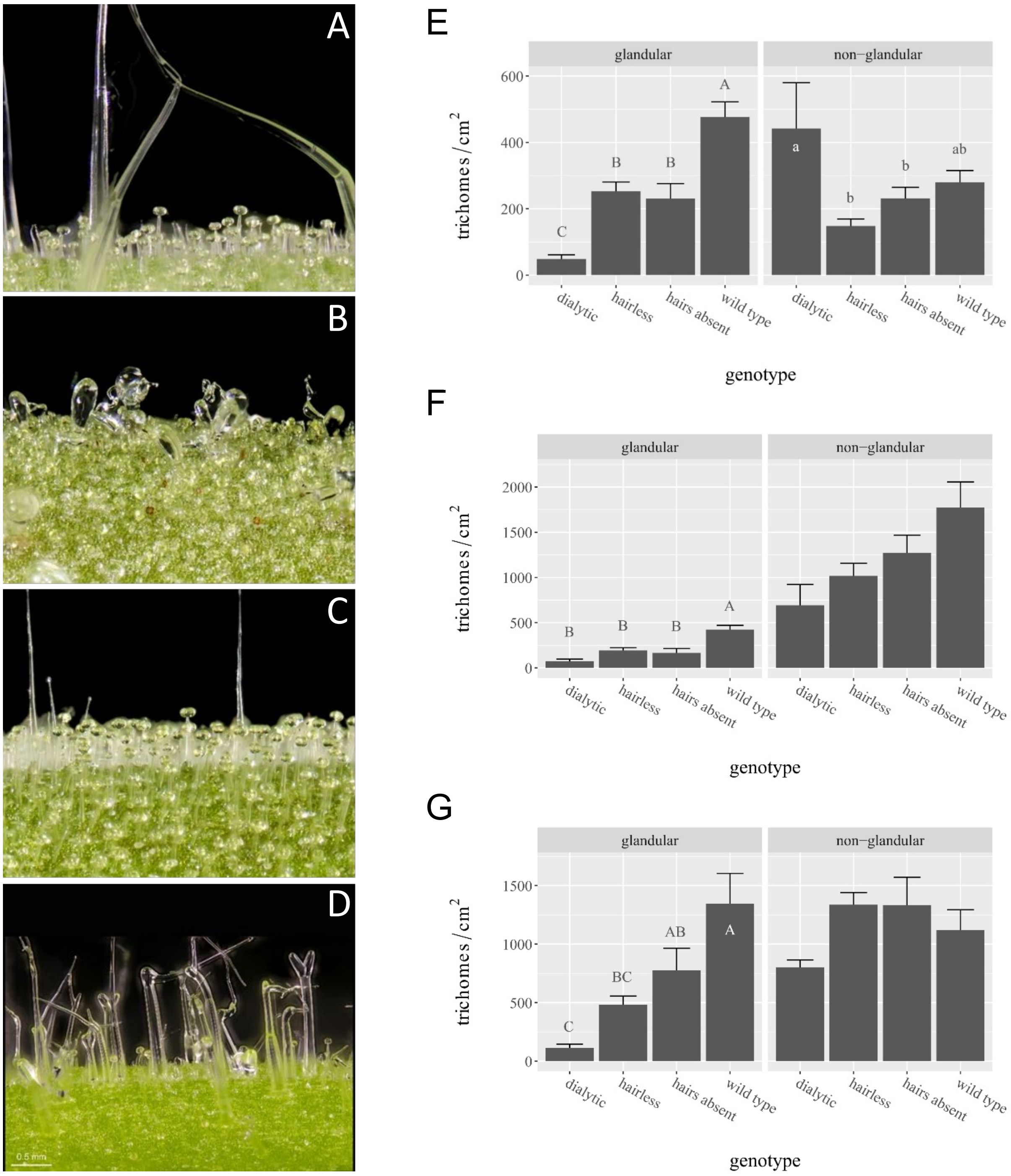

AS1 Figure 1. Close-ups of the stem surface of the wild type tomato (A) and the four tomato trichome mutants *hairless* (B), *hairs absent* (C), and *dialytic* (D). Panels E to G show densities of glandular and non-glandular trichomes estimated from the upper side (E), underside of leaves (F) and the stem (G) of the plant. Different letters indicate significant differences in trichome densities

within each panel (contrasts after GLM), a – c for non-glandular trichomes, A – C for glandular trichomes. A - D kindly provided by Jan van Arkel (University of Amsterdam, IBED).

References

Glas, J. J., Schimmel, B. C. J., Alba, J. M., Escobar-Bravo, R., Schuurink, R. C., & Kant, M. R. (2012). Plant glandular trichomes as targets for breeding or engineering of resistance to herbivores. *International Journal of Molecular Sciences, 13,* 17077–17103. https://doi.org/10.3390/ijms131217077

Kang, J. H., Shi, F., Jones, A. D., Marks, M. D., & Howe, G. A. (2010). Distortion of trichome morphology by the *hairless* mutation of tomato affects leaf surface chemistry. *Journal of Experimental Botany, 61*(4), 1053–1064. https://doi.org/10.1093/JXB/ERP370

Lenth, R. (2016). Least-squares means: the R package lsmeans. *Journal of Statistical Software, 69*, 1–33. https://doi.org/10.18637/jss.v069.i01
