# Supplementary material for: Enhanced top‐down control of herbivore population growth on plants with impaired defences
Source: Funct Ecol. 2022 Sep 21;36(11):2859–72. doi: 10.1111/1365-2435.14175 (PMC9826462; doi:10.1111/1365-2435.14175)
Supplement: Supplementary file 2 — Appendix S2 [file FEC-36-2859-s002.pdf]

Supporting Information for

Enhanced top-down control of herbivore population growth on plants with impaired defences

By: Legarrea S., Janssen A., Dong L., Glas J.J., van Houten Y.M., Scala A., & Kant M.R.

Published in Functional Ecology

Appendix S2: Statistical analysis of the population dynamics experiments by date

Generalized linear models were performed to gain insight into the specific dates in which treatments showed significant differences. A Gaussian distribution and an identity link function were assumed for these generalized linear models (the glm function in R (version 4.2.0 (2022-04-22 ucrt))). The dependent variable was the density of mites (calculated as average number of individuals per leaflet) and the fixed factor was the treatment (i.e. a combination between plant genotype and the presence of predators). Pairwise comparisons were adjusted with Tukey (α = 0.05) using the package "emmeans" (Lenth, R., Singmann, H., Love, J., Buerkner, P., & Herve, M. (2019). Estimated Marginal Means, aka Least-Squares Means v. 1.1.).

AS2 Table 1. Densities per date of adapted spider mites (*Tetranychus urticae)* in the population dynamics experiments shown in Figure 3a

**Treatment[a]**	**Predator**	**Genotype**	**Density of spider mites per leaflet (mean ± SE)***									
			**day 14**		**day 21**		**day 28**		**day 35**		**day 42**	
**Hairless - pred**	**no**	**Hairless**	15.3 ± 4.3	ns	121.5 ± 37.5	ns	130.1 ± 29.8	a	39.4 ± 33.6	a	3.8 ± 2.5	a
**Wild type - pred**	**no**	**Wild type**	14.2 ± 4.0	ns	107.8 ± 21.3	ns	108.9 ± 19.4	a	20.9 ± 7.5	a	4.4 ± 2.7	a
**Hairless + pred**	**yes**	**Hairless**	13.2 ± 1.9	ns	40.1 ± 7.9	ns	0.2 ± 0.1	b	0.05 ± 0.03	b	0.05 ± 0.03	b
**Wild type + pred**	**yes**	**Wild type**	11.6 ± 3.1	ns	90.8 ± 46.7	ns	40.7 ± 36.5	ab	2.4 ± 2.02	b	0.9 ± 0.6	ab

*Different letters in columns indicate significant differences after glm on log-transformed data and pairwise comparisons (Tukey, α = 0.05)
[a]Treatment refers to the two plant genotypes (Wild type and Hairless) with or without the release of predatory mites (*Phytoseiulus persimilis*).

AS2 Table 2. Densities per date of predatory mites (*Phytoseiulus persimilis*) in the population dynamics experiments shown in Figure 3b

Treatment[a]	Predator	Genotype	Density of predatory mites per leaflet (mean ± SE)*									
			day 14		day 21		day 28		day 35		day 42	
**Hairless + pred**	**yes**	**Hairless**	0.87 ± 0.32	ns	4.975 ± 3.45	ns	3.65 ± 0.73	a	0.2 ± 0.08	ns	0.6 ± 0.2	a
**Wild type + pred**	**yes**	**Wild type**	0.25 ± 0.24	ns	1.825 ± 1.26	ns	0.825 ± 0.61	b	0.1 ± 0.1	ns	0.075 ± 0.055	b

*Different letters in columns indicate significant differences after glm on log-transformed data and pairwise comparisons (Tukey, α = 0.05)
[a]Treatment refers to the two plant genotypes under study (Wild type and Hairless) with predators.

AS2 Table 3. Densities per date of maladapted spider mites (*Tetranychus urticae)* in the population dynamics experiments shown in Figure 3c

Treatment[a]	Predator	Genotype	Density of spider mites per leaflet (mean ± SE)*									
			day 14		day 21		day 28		day 35		day 42	
**Wild type + pred**	**yes**	**Wild type**	1.5 ± 0.7	ns	3 ± 2.05	ns	1.9 ± 0.7	ns	0.375 ± 0.128	a	1.2 ± 0.5	ns
**Hairless + pred**	**yes**	**Hairless**	0.6 ± 0.2	ns	0.175 ± 0.099	ns	0.55 ± 0.597	ns	0.075 ± 0.055	b	0.35 ± 0.40	ns

*Different letters in columns indicate significant differences after glm on log-transformed data and pairwise comparisons (Tukey, α = 0.05)
[a]Treatment refers to the two plant genotypes (Wild type and Hairless) with predators.

AS2 Table 4. Densities per date of predatory mites (*Phytoseiulus persimilis)* in the population dynamics experiment shown in Figure 3d

Treatment[a]	Predator	Genotype	Density of predatory mites per leaflet (mean ± SE)*									
			day 14		day 21		day 28		day 35		Day 42	
**Hairless + pred**	**yes**	**Hairless**	0.15 ± 0.06	ns	0.025 ± 0.029	ns	0.025 ± 0.029	ns	0.025 ± 0.029	ns	0	ns
**Wild type + pred**	**yes**	**Wild type**	0.05 ± 0.03	ns	0	ns	0.025 ± 0.029	ns	0	ns	0	ns

*Different letters in columns indicate significant differences after glm on log-transformed data and pairwise comparisons (Tukey, α = 0.05)
[a]Treatment refers to the two plant genotypes under study (Wild type and Hairless) with predators.

AS2 Table 5. Densities per date of tomato russet mites (*Aculops lycopersici)* in the population dynamics experiment shown in Figure 4a

Treatment[a]	Predator	Genotype	Density of tomato russet mites per leaflet (mean ± SE)*													
			day 14		day 21		day 25		day 28		day 33		day 36		day 39	
**Wild type - pred**	**no**	**Wild type**	54.7 ± 9.3	ns	119.1 ± 20.5	a	190.3 ± 25.4	a	317.7 ± 34.6	a	276.0 ± 28.6	a	369.3 ± 36.7	a	541.1 ± 142.5	a
**Wild type + pred**	**yes**	**Wild type**	49.6 ± 14.7	ns	132.4 ± 8.7	a	197.9 ± 5.8	a	234.0 ± 20.9	a	231.3 ± 34.7	a	360.7 ± 67.6	a	273.3 ± 84.2	a
**Hairless - pred**	**no**	**Hairless**	45.9 ± 13.4	ns	91.1 ± 14.3	a	59.3 ± 16.8	b	80.7 ± 12.8	b	32.7 ± 6.2	b	62.0 ± 9.1	b	80.7 ± 16.0	b
**Hairless + pred**	**yes**	**Hairless**	43.1 ± 12.3	ns	49.3 ± 4.0	b	40.1 ± 6.3	b	60.4 ± 14.1	b	31.7 ± 11.1	b	61.0 ± 9.8	b	50.0 ± 3.1	b

*Different letters in columns indicate significant differences after glm on log-transformed data and pairwise comparisons (Tukey, α = 0.05)
[a]Treatment refers to the two plant genotypes (Wild type and Hairless) with or without the release of predatory mites (*Amblydromalus limonicus*).

AS2Table 6. Densities per date of predatory mites (*Amblydromalus limonicus*) in the population dynamics experiment shown in Figure 4b

Treatment[a]	Predator	Genotype	Density of predatory mites per leaflet (mean ± SE)*						
			day 14	day 21	day 25	day 28	day 33	day 36	day 39
**Hairless + pred**	**yes**	**Hairless**	0.16 ± 0.05 ns	0.4 ± 0.1 a	0.17 ± 0.11 ns	0.36 ± 0.12 a	1.9 ± 0.55 ns	3.3 ± 1.0 ns	2.7 ± 0.8 ns
**Wild type + pred**	**yes**	**Wild type**	0.16 ± 0.06 ns	0.1 ± 0.1 b	0.04 ± 0.05 ns	0 b	0.27 ± 0.22 ns	1.2 ± 0.9 ns	3.8 ± 2.8 ns

**Different letters in columns indicate significant differences after glm on log-transformed data and pairwise comparisons (Tukey, α = 0.05)
[a]Treatment refers to the two plant genotypes (Wild type and Hairless) with predators.
