# Supplementary material for: Enhanced top‐down control of herbivore population growth on plants with impaired defences
Source: Funct Ecol. 2022 Sep 21;36(11):2859–72. doi: 10.1111/1365-2435.14175 (PMC9826462; doi:10.1111/1365-2435.14175)
Supplement: Supplementary file 3 — Figure S1 [file FEC-36-2859-s003.pdf]

Supporting Information for

Enhanced top-down control of herbivore population growth on plants with impaired defences

By: Legarrea S., Janssen A., Dong L., Glas J.J., van Houten Y.M., Scala A., & Kant M.R.

Published in Functional Ecology

Figure S1: Conceptual figure on the experimental approach

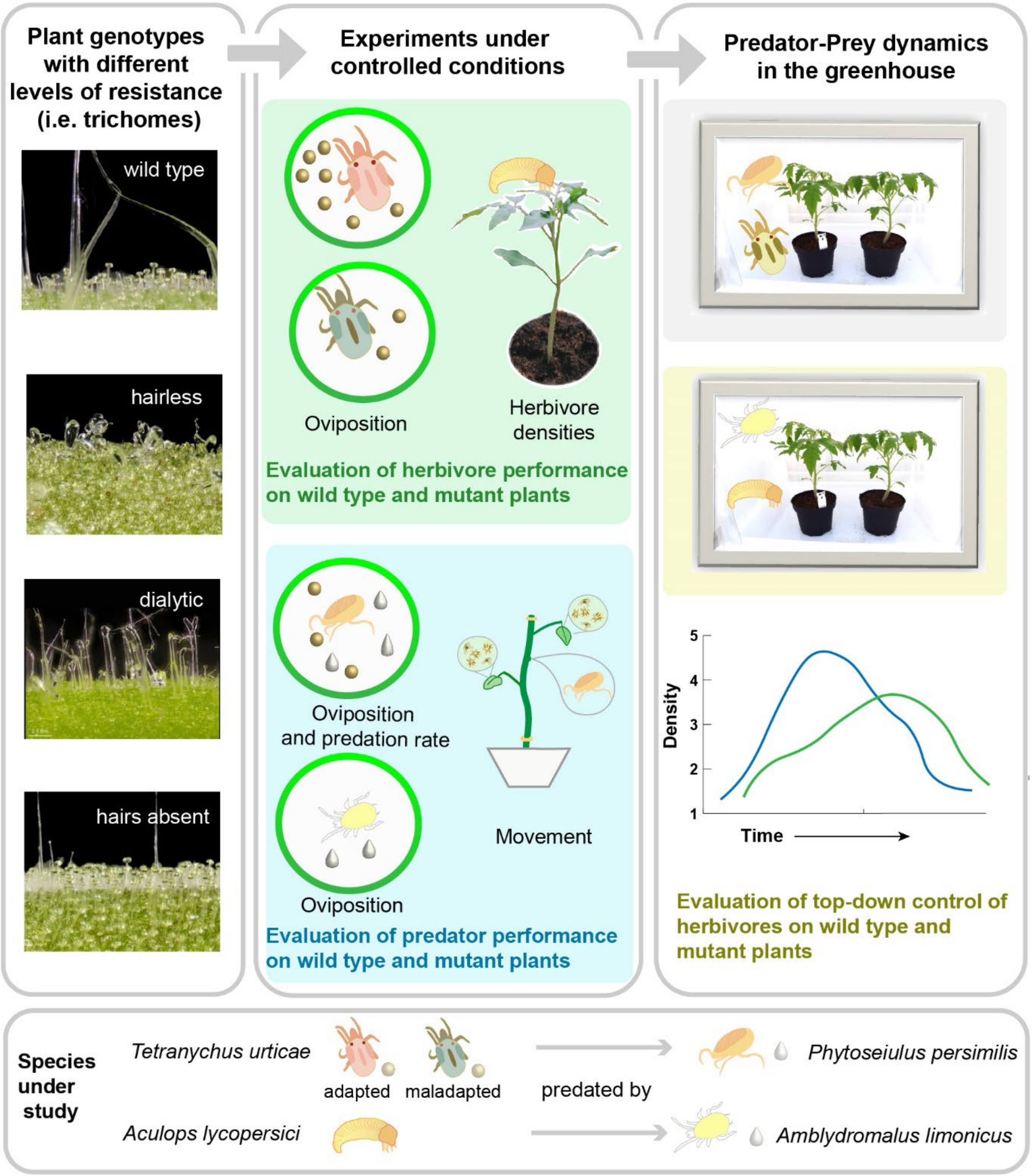

Fig. S1. Conceptual figure representing the experimental overview, from the materials chosen (i.e. plant genotypes and arthropod species under study), to icon representation of the experiments conducted under laboratory conditions and population dynamics experiments in the greenhouse.
